# Techno-Economic Assessment of a Closed-Loop Circular Economy for Polylactic Acid

**DOI:** 10.1021/acssuschemeng.5c01154

**Published:** 2025-07-15

**Authors:** Rongrong Zhang, Shuya Jia, Jun Li, Yong Xu, Hsinghung Chen, Xiaolei Zhang

**Affiliations:** † The Institute for Sustainable Development, 58816 Macau University of Science and Technology, Macau 999078, China; ‡ Department of Chemical and Process Engineering, 3527 University of Strathclyde, Glasgow G1 1XJ, U.K.; § Jiangsu Co-Innovation Center of Efficient Processing and Utilization of Forest Resources, College of Chemical Engineering, Nanjing Forestry University, Nanjing 210037, People’s Republic of China

**Keywords:** polylactic acid (PLA), tech-economic analysis, circular economy, polymer recycling, sustainable production

## Abstract

As the global demand for polylactic acid (PLA), a biodegradable plastic, continues to increase, both its production and consumption have been increasing steadily each year. This growth poses challenges for managing PLA waste disposal, highlighting the need for innovative solutions. In this study, techno-economic simulations were performed to recycle waste PLA using three distinct processes across seven different scenarios. These processes not only address waste management issues but also promote a transition from a linear to a circular plastic economy. The findings demonstrate that, compared to conventional industrial processes, the proposed recycling method using a TiO_2_/SiO_2_ catalyst has significantly increased PLA yield by 59.87% while reducing costs by 22.87%. The primary factor driving this improved yield is the choice of catalyst, which plays a critical role in determining the activity and selectivity of the direct conversion of methyl lactate to lactide in the gas phase. This research provides a sustainable and economically viable solution for recycling PLA waste, aligning with the growing demand for environmentally friendly alternatives in the plastics industry. The findings are essential for advancing the development of a circular plastics economy and emphasize the importance of catalyst optimization in improving the efficiency and sustainability of PLA recycling processes.

## Graphical abstract

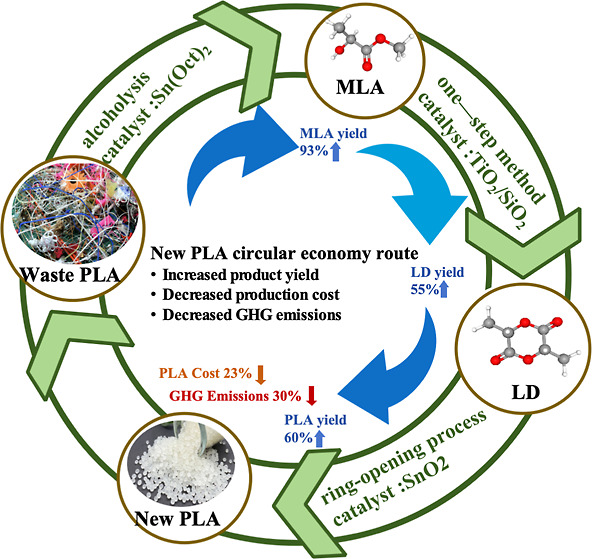

## 1 Introduction

Global production capacity for biodegradable plastics is projected to grow significantly, increasing from approximately 2.47 million tonnes in 2024 to around 5.73 million tonnes in 2029. Among these plastics, Polylactic acid (PLA) is the most widely used, accounting for 37.1% of all biodegradable plastics produced in 2024, with its share expected to rise to 42.3% by 2029.^1^ PLA is a well-known biodegradable polymer with applications ranging from 3D printing and fiber to packaging and is environmentally friendly, with a carbon footprint that is only 20–30% that of traditional plastics and fibrous materials.^2^ However, due to its slow biodegradation and the lack of overall recycling strategies, large-scale use of PLA could potentially become a significant source of future plastic contamination.^3^

While PLA is valued for its biodegradability, its natural degradation process is relatively slow and influenced by various environmental factors such as temperature, humidity, and microbial activity.^2,4,5^ Under industrial composting conditions, the decomposition of PLA waste can result in the loss of its embedded energy, and the potential leaching of additives may negatively impact soil quality.^2^ These issues underscore the need for effective management strategies for discarded PLA. Naturally, PLA takes up to one year to degrade at 20 °C, with faster degradation occurring within 12 weeks at higher temperatures (e.g., >25 °C), which presents practical challenges. The accumulation of waste PLA (WPLA), whether through stockpiling or landfilling, not only occupies valuable space but also poses environmental risks. Additionally, the end products of PLA degradation, primarily carbon dioxide and water, are not suitable for direct recycling or reuse, leading to a significant waste of resources.^6^ To address these challenges and advance a circular plastic economy, PLA waste must be managed carefully through improved recycling and innovative disposal methods.

Currently, three main pathways being employed for PLA recycling are mechanical recycling, chemical recycling, and thermal recycling (e.g., pyrolysis). Mechanical recycling involves processing WPLA into a lower-grade product through mechanical methods such as screw extrusion, injection molding, and blow molding.^7,8^ This approach offers several advantages, including low treatment costs, reduced greenhouse gas (GHG) emissions, low consumption of nonrenewable energy, and decreased environmental acidification. For the treatment of WPLA, reuse should be the first choice followed by mechanical recycling. For those low-grade WPLA, which cannot be mechanically recycled, chemical recycling should be considered.^7^ Chemical recycling involves the chemical breakdown and reprocessing of plastic waste into monomers or other valuable materials using techniques such as hydrolysis^9,10^ and enzymatic degradation.^11^ Pyrolysis, which refers to the thermal decomposition of plastic waste in an inert environment, is a method for recycling of both energy and materials.^7^ PLA pyrolysis presents challenges, such as the difficulty in obtaining high-purity products and the need for higher temperatures (300–700 °C),^4,7,12^ which can lead to numerous side reactions and the formation of complex byproducts. Additionally, the process generates large amounts of waste heat and requires extra energy inputs, which may limit its efficiency and cost-effectiveness.^13^ Compared to the other two recycling pathways, chemical recycling stands out as a key method because it offers the opportunity to produce value-added materials and enables a circular polymer production economy since recovered virgin monomers can be repolymerized allowing for an indefinite amount of recycles.^14^

The chemical recycling of PLA typically involves the conversion of WPLA into lactide (LD) through hydrolysis or alcoholysis,^15^ followed by the ring-opening polymerization (ROP) of LD, resulting in high molecular weight PLA with relatively uniform chain lengths. This controlled recycling process not only ensures the production of PLA with desirable properties but also facilitates a circular economy by enabling the recycling of waste polymer back into a new polymer.^16^ Among the chemical recycling of PLA, the two-step LD synthesis is the classical and industrial practiced method of preparing LD.^17,18^ The lactic acid (LA) monomer initially underwent polycondensation, resulting in the formation of LA oligomers through an autocatalytic esterification reaction. Then, the LA oligomers were purified by catalytic depolymerization reaction to obtain LD. Unfortunately, current LD production is time-consuming and of low purity due to the racemization, which is a major obstacle to achieving cost-effective PLA.

This problem can be solved by an alternative method for LD production based on the gas-phase transesterification of methyl lactate (MLA), providing a direct one-step synthetic route to LD. The catalytic gas-phase reaction in the fixed-bed reactors enhances performance and efficiency.^19,20^ In the gas-phase reaction, titanium-silica catalysts (e.g., TiO_2_/SiO_2_ and TiO_2_/MCM-41) showed a high LD selectivity of 88–92% at conversion rates below 50%, significantly reducing racemization. While TiO_2_ is a widely used commercial catalyst, its effectiveness in LD production is limited, with selectivity typically below 50%.^19^ Titanium-silica catalysts feature covalent Ti–O–Si bonds between the Ti-active sites and the silica support. These covalent bonds prevent TiO_2_ leaching from the support and improve the interaction between the active sites and the support, thereby enhancing catalytic performance.^21,22^ Two types of SiO_2_ supports are used: amorphous SiO_2_ and ordered mesoporous MCM-41. The amorphous SiO_2_ is chosen for its high specific surface area, which enhances the catalytic activity by increasing the number of active sites available for the reaction. The high specific surface area and ordered pore structure of MCM-41 allow for better dispersion of TiO_2_ particles, resulting in improved catalytic performance.^23^ The ordered mesoporous structure of MCM-41 also aids in the diffusion of reactants and products, which can enhance the reaction kinetics.

To evaluate the technical and economic performance of the PLA recycling process, three distinct processes across seven different scenarios were constructed in this research. For all processes, we selected the WPLA as the raw material for recycling,^24^ and before chemical recycling, WPLA was pretreated to separate out impurities. The three processes to be investigated in this research are hydrolysis (Process 1), alcoholysis (Process 2), and alcoholysis with MLA recycling (Process 3). In total, seven scenarios based on different catalytic systems were considered. The overall aim of this research is to enhance the sustainability and economic viability of the PLA recycling process.^25^ To achieve this, we performed techno-economic analysis, GHG emissions analysis, and energy analysis on the seven scenarios using Aspen Plus.^26^ Furthermore, sensitivity analyses are performed to evaluate the influence of key factors, including feedstock, process parameters, and economic parameters, on economics and sustainability.

## 2 Methodology

### 2.1 Pretreatment of WPLA

Postconsumer WPLA is typically affected by the presence of additives and contamination from other materials in the waste phase.^27,28^ In most cases, plastic additives are not chemically bonded to the polymer chains, allowing them to leach from the plastic products under certain conditions. Additionally, the additives are present in relatively small amounts and do not significantly affect the overall chemical behavior of PLA during the recycling processes.^29,30^ As illustrated in Figure 1, the recycling process of WPLA mainly includes the following steps:^30–32^ (1) collection and transportation of WPLA to the treatment plant, (2) pretreatment of WPLA to remove additives and contaminations, and (3) chemical recycling processes. After collection, postconsumer PLA waste is transported to a treatment facility for sorting with near-infrared (NIR)-based sorting methodologies.^28^ In subsequent steps, the sorted PLA waste is washed with a 2% aqueous NaOH solution at 70 °C and then dried. This washing step helps to remove residual impurities that could not be separated in previous steps, reducing the impurity content to less than 5%. Consequently, a high-purity WPLA feedstock suitable for chemical recycling is obtained; the dried WPLA is then shredded and separated, completing the pretreatment process.

**Figure 1:**
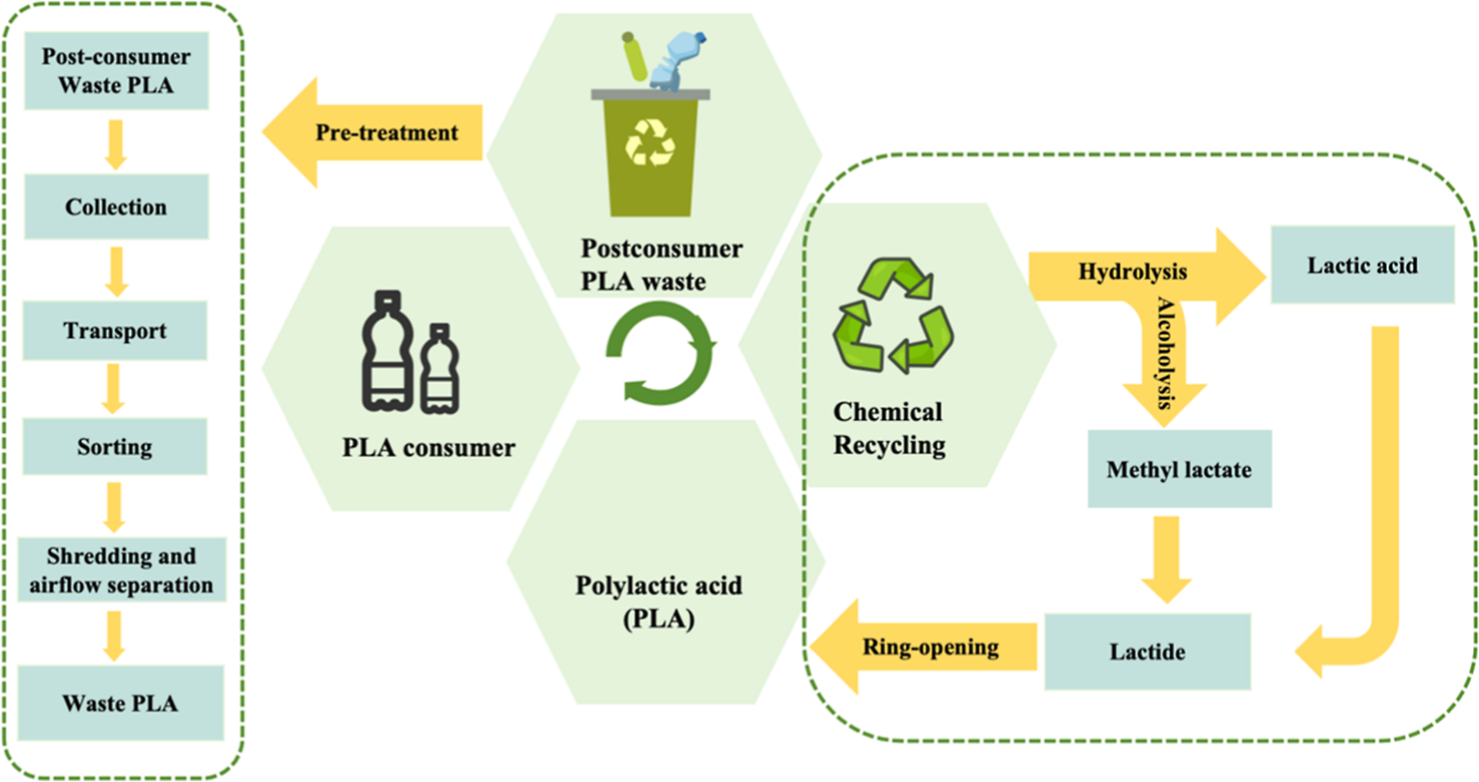
Process flow of a closed-loop circular recycling for PLA.

### 2.2 Three PLA Recycling Processes

Three distinct processes for the conversion of WPLA into valuable polymer products were investigated in this work, as shown in Figure 2. Process 1 involves the hydrolysis of WPLA to LA, which is then polymerized through a two-step process to form LD.^33^ This intermediate then undergoes ROP for the synthesis of the end product, newly formed PLA.^34^ Process 2 diverges with the alcoholysis of WPLA to produce MLA, which is directly converted to LD in a one-step gas-phase reaction.^35,36^ Mirroring Process 1, the LD then undergoes ROP to form PLA. Notably, the different catalysts TiO_2_/SiO_2_, TiO_2_/MCM-41, and TiO_2_ were used in LD production and defined as process 2a, 2b, and 2c.^18^ Process 3 is an improvement over Process 2. In Process 3, the synthesis of MLA and PLA mirrors that of Process 2, with the recycling of unreacted MLA was added to enhance the yield of LD because the conversion efficiency of MLA to LD is suboptimal.^36–38^ Additionally, Process 3 is further differentiated by the employment of distinct catalytic systems:^18^ TiO_2_/SiO_2_, TiO_2_/MCM-41, and TiO_2_, which are, respectively, termed Process 3a, 3b, and 3c.

**Figure 2:**
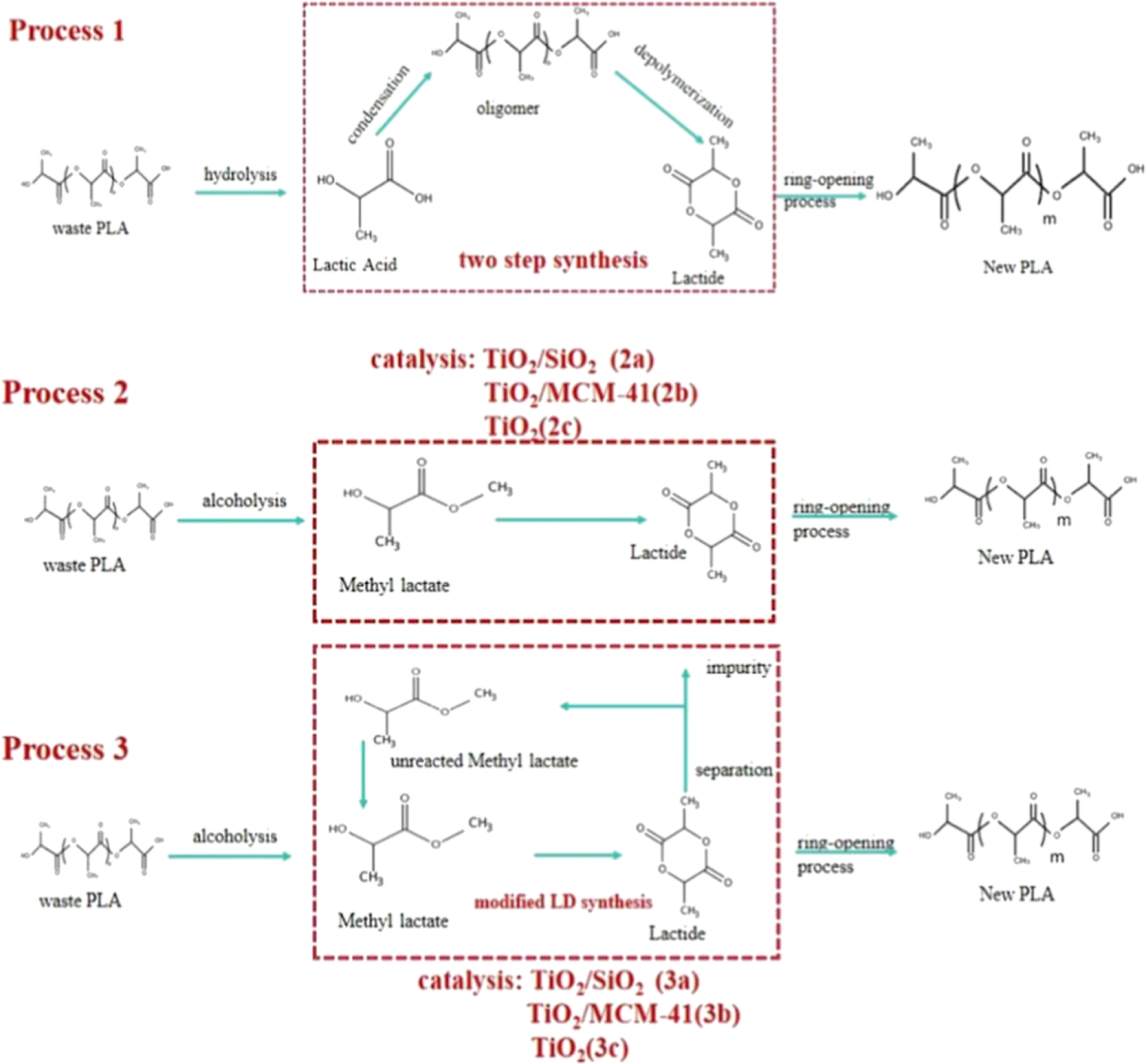
Processes 1, 2, and 3 of cycle-closed PLA waste treatment options.

### 2.3 Model Details

The process models for the three synthesis processes were built using Aspen Plus adopting the NRTL (nonrandom two-liquid) thermodynamic model. The missing parameters of PLA and WPLA were estimated by the UNIFAC group contribution methods.^39,40^ The principal distinction between WPLA plastics and newly manufactured PLA products lies in the chain length, or more precisely, the molecular weight.^41^ The reaction yield and selectivity data, crucial for evaluating the efficiency and specificity of the reactions, have been extracted from relevant literature and integrated into the process simulations.^33,42^ We assume that all processes are designed to treat the same amount of WPLA, 20,000 kg per h, to ensure a fair comparison.

The baseline scenario for the WPLA chemical recycling is Process 1, hydrolysis of WPLA followed by a two-step process to form LD, and its reactor operation is shown in Figure 3, Tables 1, and Tables S1, S2. A2, A4, A6, and A9 represent distinct types of reactors, with A6 designated as the depolymerization reactor, while the others function as restoration reactors.^43^ A3, A5, A7, and A10 serve as flash tanks designed to eliminate impurities, and A8 is a distillation column engineered to achieve a higher purity of LD. In Process 1, WPLA and water are introduced into the reactor (A2) at a temperature of 180 °C for a duration of 1.5 h,^12,26,44^ resulting in a LA solution with an impressive conversion rate of 85.9%. This efficient conversion is facilitated by a rational mechanism proposed by Wanhua Wu^45^ for the Diphenyl phosphate (DPP)-catalyzed hydrolysis of PLA.^33^ DPP is an economically viable commercial acidic organocatalyst, priced in the range of $70–150 per kilogram. The dual activation of PLA and water by DPP enables the scission of the ester bond along the PLA chain, thereby initiating a rapid and stochastic hydrolysis of PLA. This mechanism is akin to the bifunctional activation observed in reactions such as the ROP of cyclic esters catalyzed by DPP.^46^

**Figure 3:**
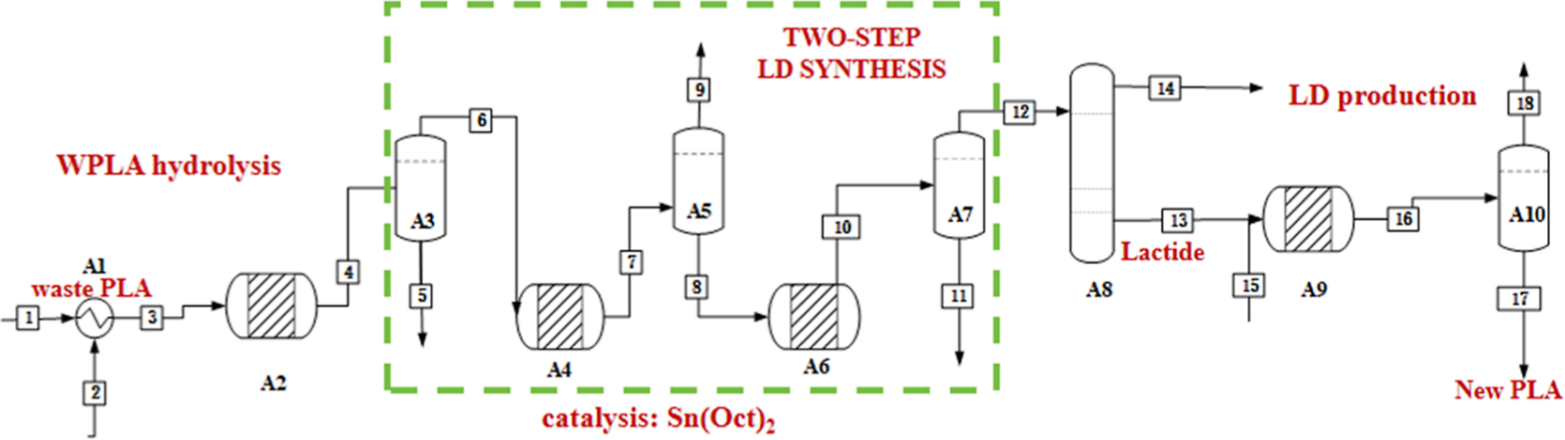
Flow diagram and key reaction steps of Process 1.

**Table 1:** Key Parameters for Process 1

variable	value	variable	value
A2 temperature	180 °C^47^	A4 temperature	200 °C^52^
A2 pressure	1 bar^45^	A4 pressure	0.33 bar^50^
A3 temperature	200 °C^52^	A6 pressure	0.01 bar^26^
A3 pressure	0.33 bar^50^	A8 stage	30 ^12^
A5 temperature	220 °C^52^	A9 temperature	80 °C^46^
A5 pressure	0.01 bar^50^	A9 pressure	1 bar^44^
oligomer PLA	594g/mol^52^	A10 temperature	220 °C^54^
catalyst	Sn(Oct)_2_,0.2 wt %	A10 pressure	0.1 bar^52^

In the current industrial production of LD, the traditional process commences with the removal of water from LA in a flash vessel, A3, to generate a prepolymer with a low molecular weight, known as an oligomer.^47^ The second step involves the catalytic depolymerization of the oligomer within reactor A6, leading to the formation of cyclic dimers, specifically LD. Following its formation, LD is refined through distillation to achieve the desired purity levels. In Process 1, reactor A4 promotes lactate dehydration and polymerization and operates at low pressure (0.07–0.33 bar) and high temperature (200 °C), while oligomers are formed by an autocatalytic esterification reaction. Since the average molecular weight of the oligomer affects the output of the depolymerization reactor (A6), the average molecular weight of the lactic acid oligomer (Mn) increases with the reactor temperature,^48^ so the temperature of A4 needs careful control. Furthermore, the total yield of the oligomer was varied in the range of 80–95%.^5^ The liquid phase of LA and LA oligomers containing minor impurities is then fed into the depolymerization reactor (A6), where the LA oligomer forms lactides by catalytic depolymerization. The reaction raw material is pure l-lactic acid, and l-lactide (L-LD) and meso-lactide (M-LD) are formed under the vacuum pressure of 200–240 °C and 10–50 Torr.^49^ The reaction catalysts is Sn­(Oct)_2_,^50^ which can improve the conversion rate of the depolymerization reaction and the selectivity of L-LD relative to M-LD. The unreacted PLA oligomers were then removed by a flash reactor (A6). The remaining stream flows into purification tower A8, which operates with 30 stages and a reflux ratio of 15. Then, L-LD and water enter the reactor (A9) to further generate the PLA, and 100% of the final product PLA is separated by different boiling points.^6,8,51^

In Process 2 and Process 3, PLA is depolymerized by alcoholysis to produce value-added products. B2 is designated as the depolymerization reactor, while B5 and B8 function as restoration reactors. The conversion rates for each step are confirmed according to the literature. B3, B6, and B9 serve as flash tanks for impurities removal, while B7 is a distillation column engineered to achieve a higher purity of LD (Key parameters are provided in Tables S4 and S5). The reactor’s operation is delineated in the conditions as depicted in Figure 4 and Table 2. The conversion of PLA waste into alkyl lactate or lactate by alcoholysis has the following advantages: high added value, high yield of lactate, simple purification, and no need for the removal of water due to the fact that it is in a liquid state at room temperature. Under relatively mild reaction conditions, the PLA waste product will be converted into MLA by transesterification reaction.^38^ As shown in Figure 4, the mix feed (WPLA and methanol) is first sent to a reactor B2 to synthesize MLA. The degradation of PLA by Zinc­(II) Octoate was also carried out under solvent-free conditions,^53^ with a catalyst loading of 16 wt %.^35^ In this case, it was necessary to use a temperature of 130 °C to completely dissolve the polymer into MeOH. However, the stream 24 is sent to a flash B3 to remove water and impurity, and the evaporated MLA (stream 25) is mixed with a nitrogen stream (1.19 kg/h, 1.00 bar) in the mixer (B4).^54,55^

**Figure 4:**
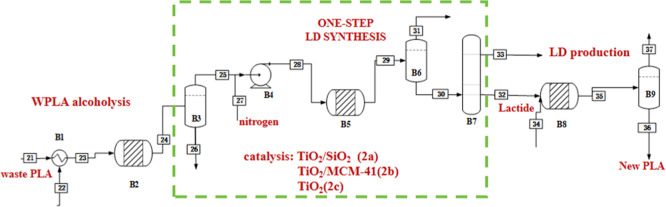
Process 2a, 2b, and 2c flow diagram of the proposed PLA synthesis.

**Table 2:** Key Parameters for Process 2 and Process 3

variable	value	variable	value
B2 temperature	130 °C^60^	B6 temperature	70 °C^20^
B2 pressure	1 bar^56^	B6 pressure	0.1 bar^20^
B3 temperature	140 °C^61^	B7 lactide product	0.99 ^52^
B3 vapor	0.999 ^61^	B7 stage	30 ^52^
B5 reaction number	6 ^61^	B8 temperature	80 °C^15^
B5 temperature	220 °C^61^	B8 pressure	1 bar^15^
catalyst	TiO_2_/SiO_2_, TiO_2_/MCM-41, TiO_2_	B9 temperature	220 °C^62^
catalyst wt %	5%^19^	B9 pressure	0.1 bar^57^

The mixed stream (stream 28) is then fed into a reactor (B5) containing a 5 wt % catalyst. The catalysts to be considered are TiO_2_/SiO_2,_ TiO_2_/MCM-41, and TiO_2._ The catalytic reaction was performed in a custom piston flow fixed bed reactor equipped with six separate parallel quartz reactors (480 mm in length and 4 mm in inner diameter). In typical reactions, the quartz reactor is filled with 10–300 mg of catalyst loaded by quartz asbestos (sieve 250–500 μm). Before each reaction, the catalyst was pretreated at 300 °C for 1 h at a N_2_ airflow at a rate of 20 mL per minute. After pretreatment, the reactor was cooled to the desired reaction temperature. The reactor temperature should be suitably adjusted to 220 °C to ensure that the reaction occurs smoothly. The detailed reaction is shown in Figure S3. Then, the reactive stream (stream 29) enters the 70 °C, 0.1 bar flash B6, and the purpose of this step is to separate methanol from the high boiling compound. The separation system was designed considering that the compounds involved in the process have very different boiling points, methanol has a moderate boiling point (64.7 °C); M-LD and L-LD have very high boiling points of 230 °C.^8^

The fact that the conversion rate of MLA to LD was only 42% in Process 2^19^ will cause a waste of raw materials, and this issue will be improved in Process 3, as shown in Figure 5, to reuse the unreacted MLA recycle back into the reactor (Key parameters are provided in Tables S9 and S10). Then, the reactor outlet (stream 54) enters the purification column B7 with a reflux ratio of 6.11, and after a series of reactions, it yields an L-LD of 99% purity.^16^ In this process, a coolant with a lower inlet temperature is highly desirable because the reaction outlet can use a lower temperature to reduce the M-LD reaction, producing a 99% purified LD product as the bottom (stream 55).

**Figure 5:**
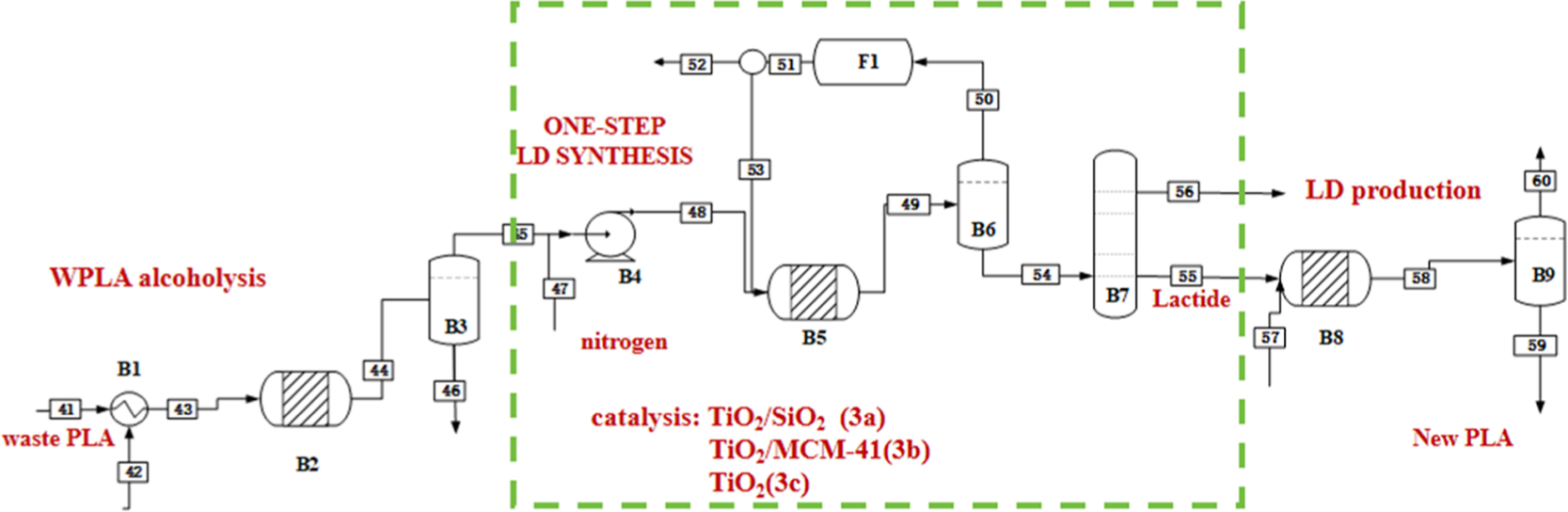
Process 3a, 3b, and 3c flow diagram of the proposed modified one-step PLA synthesis.

### 2.4 Techno-Economic Analysis

The economic performance of three different PLA synthesis processes was studied through the estimation of capital costs and operating costs of pretreatment and chemical recycling process.^58^ To establish an economic analysis, a multitude of input parameters was utilized in the study, as illustrated in Table 3.^58,59^ All data and financial assumptions used in this study came from extensive reviews of the literature, journals, and reports from academic institutions, governments, and private companies.

**Table 3:** Summary of the Economic Parameters in Production Cost Estimation

items	estimation assumption	data source
plant location	China	assumed
design on-stream factor	365 day*24 h*0.95	assumed
reference year	2024	assumed
catalytic	different catalysts of different scenario	assumed
capital cost	capital cost = FCI + working capital	
Direct cost (DC)		
equipment purchase cost (EC)	Aspen plus	Measured
installation	Aspen plus	Measured
piping	0.31*EC	Mairizal et al., 2023 ^60^
instrumentation	0.43*EC	Mairizal et al., 2023 ^60^
electrical	0.10*EC	Mairizal et al., 2023 ^60^
buildings	0.15*EC	Mairizal et al., 2023 ^60^
yard improvement	0.12*EC	Mairizal et al., 2023 ^60^
service facilities	0.55*EC	Mairizal et al., 2023 ^60^
land	0.06*EC	Mairizal et al., 2023 ^60^
Indirect Cost (IC)		
engineering and supervision	0.32*EC	Mairizal et al., 2023 ^60^
construction expense	0.34*EC	Mairizal et al., 2023 ^60^
legal expenses	0.04*EC	Mairizal et al., 2023 ^60^
contractor’s fee	0.19*EC	Mairizal et al., 2023 ^60^
contingency	0.37*EC	Mairizal et al., 2023 ^60^
fixed capital investment (FCI)	FCI = DC + IC	Mairizal et al., 2023 ^60^
working capital	0.15*FCI	Mairizal et al., 2023 ^60^
**Operating cost**		
raw materials cost	the sum of feed stock cost and catalyst cost	measured
labor cost	administrative costs and labor cost	measured
utility cost	aspen plus	Aryan et al.,(2021)^9^
Fixed expenses (FE)		
startup costs	0.09*FCI	Kwan et al.,2018 ^61^
laboratory cost for QC and QA	0.15* total labor cost	Kwan et al.,2018 ^61^
equipment maintenance and repair	0.07*FCI	Kwan et al.,2018 ^61^
operating supplies	0.15*equipment maintenance and repair cost	Kwan et al.,2018 ^61^
depreciation (20 year straight line)	0.05*FCI	Kwan et al.,2018 ^61^
insurance	0.01*FCI	Kwan et al.,2018 ^61^
plant overhead costs	0.5*equipment maintenance and repair cost	Kwan et al.,2018 ^61^
distribution and marketing costs	0.02*total operating cost	Kwan et al.,2018 ^61^
research and development	0.05*total operating cost	Kwan et al.,2018 ^61^

#### 2.4.1 Total Capital Cost Estimation

The total capital cost was calculated by combining fixed capital investment (FCI) cost (FCI) and the working capital. The FCI is the direct cost (DC) and indirect cost of plant operation, including the costs of purchasing equipment, installation, piping, and other related costs. It was evaluated by the method of the percentage of delivered-equipment cost for the solid–fluid processing plant.^50^ The specific variables are detailed in Table 3. Equipment purchase and installation costs are categorized as DC, with other DC including piping, instrumentation, electrical, buildings, yard improvement, service facilities, and land.^60^ According to Heo,^50^ these variables can be calculated based on the equipment purchase cost. Similarly, indirect costs such as engineering, construction, legal expenses, contractor’s fee, and contingencies are calculated based on the equipment purchase cost as well. The annualized capital cost is then determined by multiplying the total capital cost by the capital recovery factor *A*_*i*,*n*_, which is defined in eq 1 ^50^

1 $$A_{i,n}=\frac{i{\left(1+i\right)}^{N}}{{\left(1+i\right)}^{N}-1}$$

where *i* and *N* represent the interest rate and the lifetime of the equipment. In this study, we assume the factor *i* = 8% and *N* = 20 years.^59^ The working capital, which was assumed to be 15% of the FCI, is allocated to cover the expenses of the initialization of the plant in the start-up phase, such as the costs of purchasing raw material, utilities, and testing of equipment.^51^

#### 2.4.2 Total Operating Cost Estimation

The annual operating cost was determined by summing the costs of the raw material, utilities, labor, and fixed expenses (FE). The costs for raw material and utilities were estimated by using mass and energy balances obtained from Aspen Plus. FE include maintenance costs incurred during the plant’s production and operation.^60^ Finally, the factory also needs to allocate funds for ongoing research and development.

Based on the 2023 annual report of the company, Zhejiang Hiseng Biomaterials Co., LTD, whose main business is the production and sale of PLA, the average labor cost used in the manufacturing of PLA was calculated to be $413 per week, equivalent to an hourly wage of $10.325, based on a standard workweek of 5 days with 8 h each day. Labor costs were adjusted according to varying production outputs.

#### 2.4.3 Production Cost

The production cost of PLA is defined in eq 2

2 $$PLA\;production\;cost=\frac{annual\;cost}{annual\;production\;rate\;of\;PLA}$$

where the annual production rate of PLA was estimated using the final production mass flow obtained from Aspen Plus. The annual cost was calculated as the sum of capital cost, operating cost, and FE as shown in Table 3.

### 2.5 GHG Emissions Analysis

The GHG emissions is a predominant factor among the spectrum of environmental impact categories associated with the circular recycling of PLA.^46,62,63^ Consequently, a more comprehensive approach to assess the GHG impact of the WPLA recycling process is presented in CO_2_ equivalent.^54^ The evaluation was based on the production of 1 kg of PLA as the functional unit. First, we utilize Aspen Plus to accurately calculate the carbon emissions directly associated with each chemical recycling process, covering Scope 1 and Scope 2 emissions. To further supplement the carbon emission assessment, we used Gabi databases to calculate Scope 3 emissions.^9^ This step assures to consider emissions from various indirect aspects, such as pretreatment of postconsumer PLA, transportation throughout the supply chain, and indirect emissions from upstream and downstream, as detailed in Supporting Information Table S17.

## 3 Results and Discussion

### 3.1 Mass and Energy Balance

In Process 1, a WPLA feedstock with a flow rate of 20,000 kg/h is hydrolyzed to a 96.8% LA solution with a flow rate of 21,448.68 kg/h in the presence of DPP catalyst.^17^ After a series of separation and purification steps, the oligomeric LA is converted to 82.35% L-LD and 14.52% M-LD. The two-step synthesis of LD demonstrates a higher conversion for LD compared to that of the one-step process. However, due to racemization, the selectivity of L-LD from the two-step synthesis is lower, making it challenging to achieve high-purity L-LD. This disparity is attributed to the more complex process route and the hygroscopic nature of the intermediate LD, which complicates the purification process.

In Processes 2 and 3, the WPLA feedstock with a flow rate of 20,000 kg/h is transesterified to a 95.3% MLA solution with a flow rate of 29,368.39 kg/h under the catalysis of Sn (Oct)_2_. Comparative analysis from Figure 6 indicates that Process 2b outperforms Processes 2a and 2c in terms of MLA conversion and L-LD mass fraction, with the highest L-LD yield of 69,332 tonnes/year. The catalyst structure of TiO_2_ loaded on the amorphous silica surface has been found to exhibit robust catalytic activity for the transesterification of L-MLA, with each Ti site demonstrating a high turnover frequency, thereby facilitating the production of LD with selectivity exceeding 90% and minimal racemization.^35^ Between Processes 2b and 3b, the recycling of unreacted MLA back into the reactor is observed to enhance the MLA conversion rate, albeit at the expense of L-LD selectivity. Among the seven scenarios, Process 2c exhibits the lowest yield of LD, which increases slightly in Process 3c due to MLA recycling. The catalytic performance of TiO_2_ mainly depends on its surface-active sites. However, compared to the other two catalysts, the number of active sites is smaller or the activity is lower, resulting in reduced conversion efficiency.^19^ In the simulated process in Aspen plus, all seven scenarios utilize Sn­(Oct)_2_ to catalyze the ROP of LD to form PLA. Studies suggest that the use of tin octoate as a catalyst and benzyl alcohol as an initiator in the ROP process efficiently converts LD into PLA, yielding polymers with high molecular weights.^40^ The annual PLA production for the seven scenarios is as follows: 59,542 tonnes/year (Process1); 63,828 tonnes/year, 67,675 tonnes/year, 25,899 tonnes/year (Process 2a, 2b, and 2c); and 95,188 tonnes/year, 98,081 tonnes/year, 32718 tonnes/year (Process 3a, 3b, and 3c). In terms of production efficiency, the TiO_2_/MCM-41 catalyst demonstrates the highest conversion rate and selectivity. Detailed mass balances for all processes are provided in the Supporting Information (Tables S3, S6–S8, and S11–S13).

**Figure 6:**
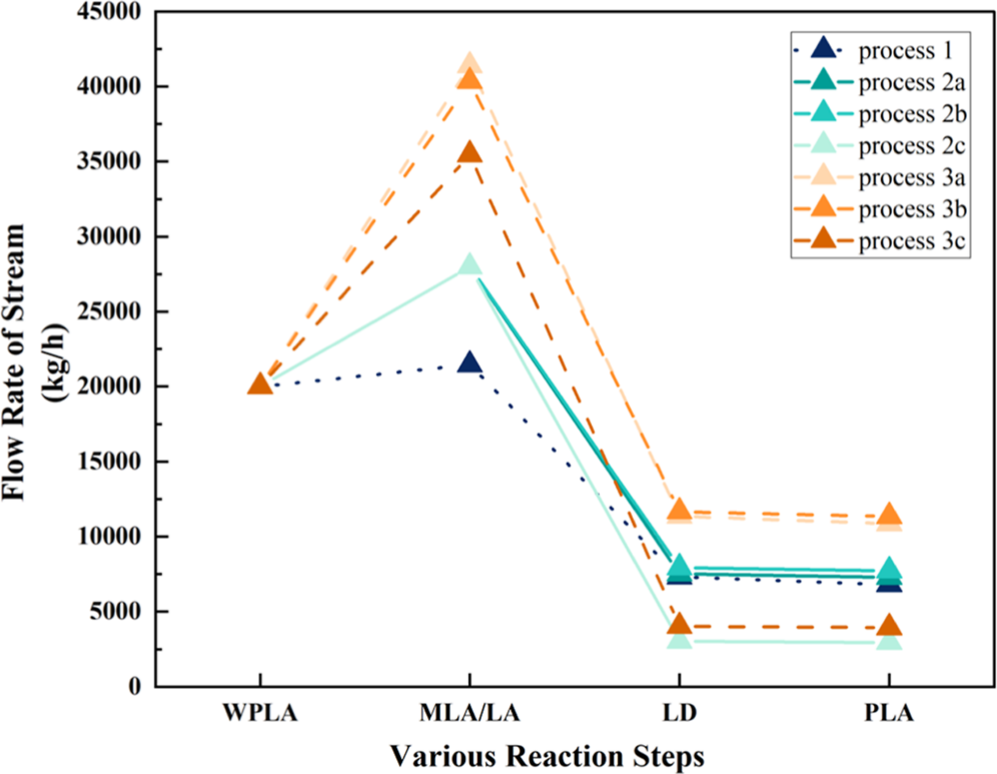
Mass balance of different processes and scenarios.

The consumption of energy for each process and scenario is clearly depicted in Figure 7. Process 1 has the highest energy consumption at 2754 billion watts per year, while Process 2c has the lowest consumption at 1417 billion watt-hours. This study calculated that in all scenarios, the purification and cooling utilities contribute the most to the total equipment utilities, reaching up to 30%. However, in the traditional two-step LD synthesis, this proportion usually exceeds 15%. Therefore, the two-step method incurs higher energy costs than other processes, and the “WPLA-MLA-LD-PLA” process is more energy-efficient. Between Process 2 and Process 3, Process 2 is more energy-efficient due to the use of fewer pieces of equipment than Process 3.

**Figure 7:**
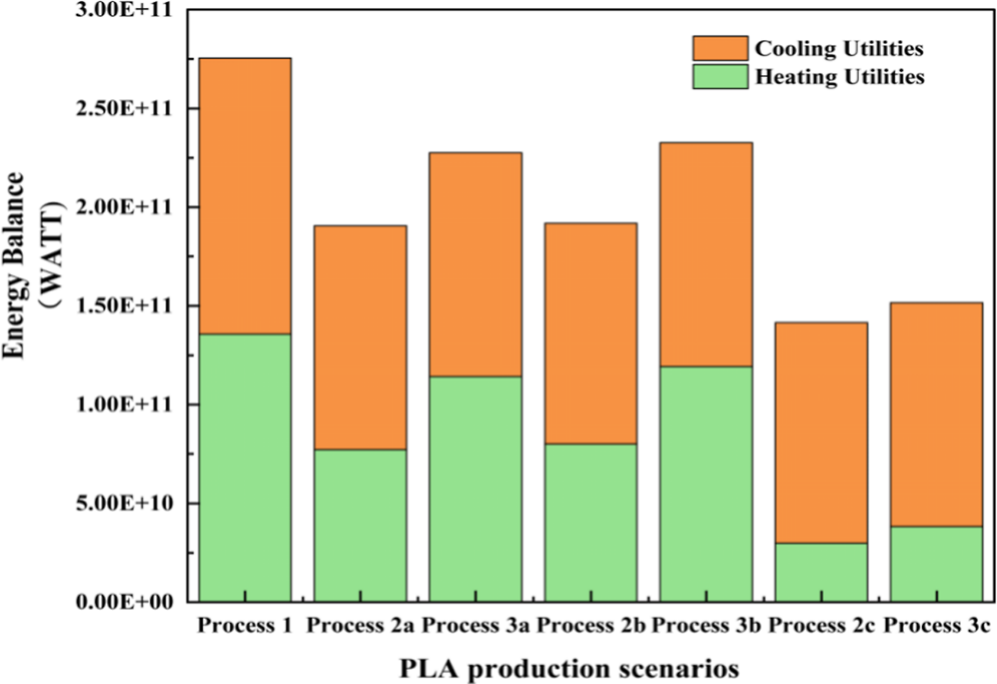
Energy balance for all the PLA production scenarios.

### 3.2 Capital Cost

Figure 8 and Table S14 present the capital cost including both FCI and working capital for all the scenarios. The annual equipment purchase cost and installation cost for Process 1 ($43.57/tonne PLA; $83.54/tonne PLA) were relatively higher than that of Process 2a ($33.77/tonne PLA; $62.85/tonne PLA) and Process 3a ($30.37/tonne PLA; $53.75/tonne PLA) since the two-step method of LD synthesis requires more equipment. Purification is the most expensive item in some technical economic studies related to the biotransformation process.^41,64^ In this study, the purification cost was calculated to contribute the most to the total equipment cost (30%) for all scenarios, however common values up to 15% in a traditional two-step LD synthesis. Therefore, Process 1 requires more refinement to obtain a higher quality L-LD. In addition to bare equipment costs, additional costs of land and plant construction are expected and summarized. The associated percentage of the cost contribution of each item to the FCI is based on the existing literature.^37^ Assuming 15% of FCI working capital is expected to cover the cost of the initial plant, such as procurement of raw materials, equipment testing and maintenance, and management and training of employees.

**Figure 8:**
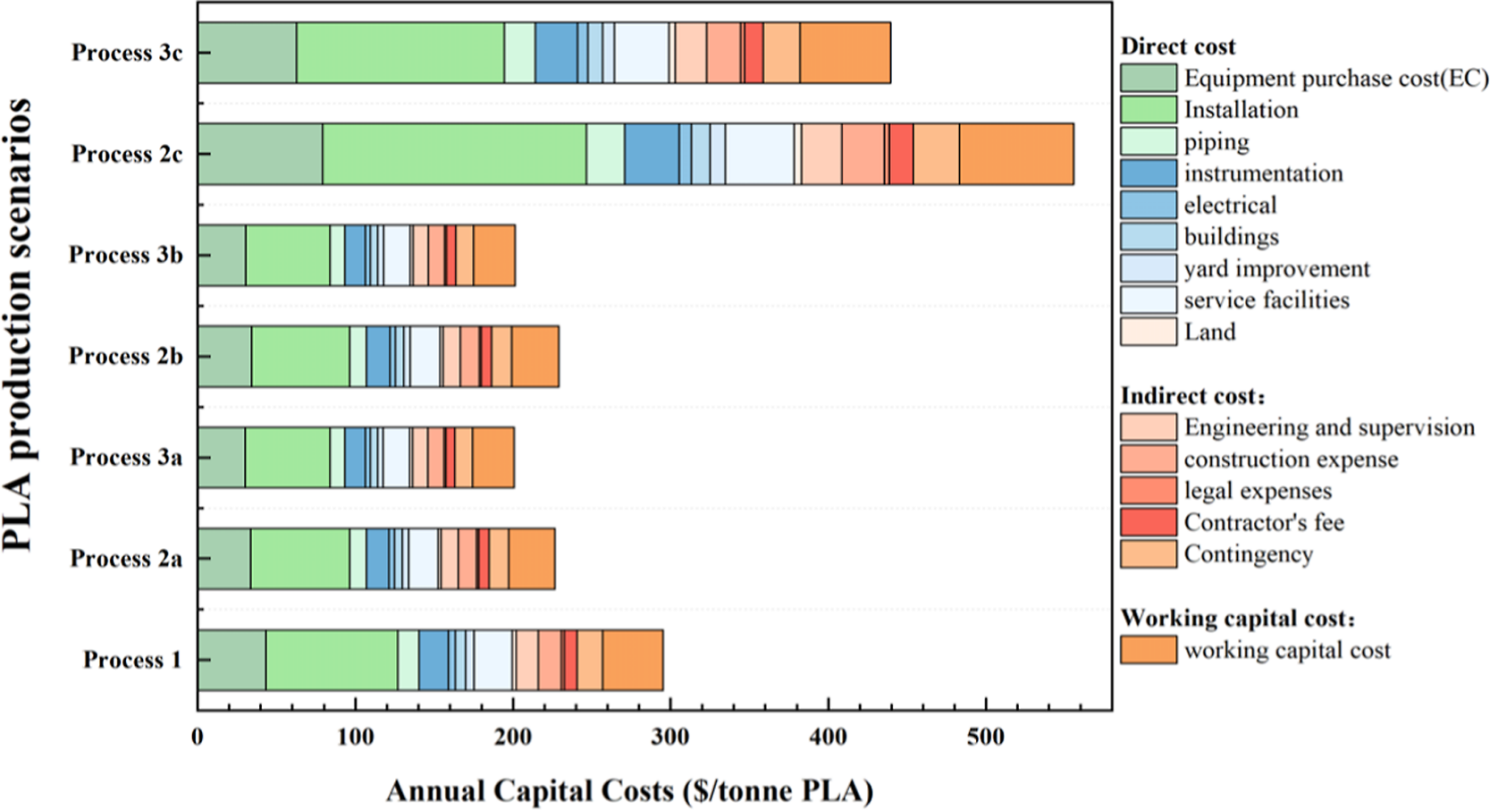
Capital costs and its breakdown for all the PLA production scenarios.

### 3.3 Operating Cost

Estimated operating costs are presented in Figure 9 and Table S15. In all scenarios, factory processing for 1 year (8322 h) had the total annual operating cost of $113,347,257/year, $105,527,222/year, $142,210,192/year, $121,708,590/year, $154,137,913/year, $140,999,004/year, and $141,963,076/year. The cost distribution of the different steps during the PLA cycle using different catalysts. It is worth noting that raw materials and utilities were the largest expenditures in Process 1, accounting for 34.31% and 41.26% of the total operating costs, respectively. Compared with processes 2a and 2b and processes 3a and 3b, two-step synthesis of LD requires more reaction equipment and procedures, more electricity, water, and requires high-pressure steam heating, the demand for two-step high-pressure steam is much greater.

**Figure 9:**
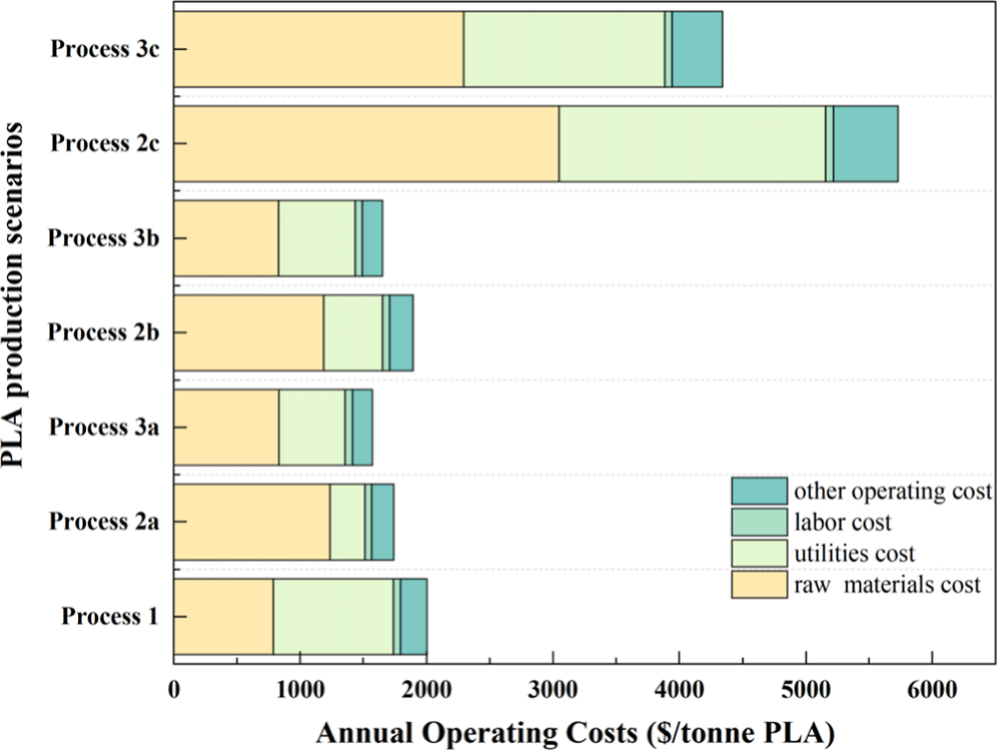
Operating costs and its breakdown for all the PLA production scenarios.

Since the one-step process involves only a single reactor, Process 2a benefits from a significant reduction in utility costs. In Process 3a, a separator is added to process 3 to recycle unreacted MLA back to the reactor for further conversion to LD. This modification leads to an increased conversion rate, a higher flow of raw materials entering the purification tower, and an increase in utility cost. For Processes 2a and 3a, Processes 2b and 3b, and Processes 2c and 3c, the difference in the price of the catalysts used in the MLA to LD synthesis process is the main reason for the different cost of the raw materials. According to preliminary calculations, the catalyst cost of Processes 2a and 3a using TiO_2_/SiO_2_ is $182 and $156, respectively, to produce 1 tonne of LD. However, the catalyst costs of Processes 2b and 3b using TiO_2_/MCM-41 are $802 and $707. The price of TiO_2_/MCM-41 is significantly higher than that of TiO_2_/SiO_2_. It is noteworthy that the catalyst costs of Processes 2c and 3c are the same as $544 because of the low conversion rate of TiO_2_ and the weight of the catalyst used in the reactor is fixed.

### 3.4 Production Cost

Advancements in recycling technologies and processes will be pivotal in achieving a sustainable and economically viable circular economy for plastics. The economic analysis for all scenarios is shown in Figure 10 and Table S16. It is evident that Processes 2c and 3c have the highest PLA production costs, driven primarily by a significant escalation in the raw material and utility expenses compared to other scenarios. Process 1, as the traditional industrial approach, has a PLA production cost of $2299/tonne. The best scenario was determined as Processes 3a and 3b, with PLA production costs of $1773/tonne and $1856/tonne, substantially reducing the production costs of PLA by 22.87% and 19.29% from the traditional industrial approach, respectively. The production in Process 3a increased from 59,542 to 95,188 tonnes/year, a 59.87% increase from the traditional industrial approach. In Process 3b, using the TiO_2_/MCM-41 catalyst, the annual production increased by 64.73% to 98,081 tonnes. The variance in cost between these two scenarios is attributed to the selection of different catalysts in Processes 2a and 2b, which influences the raw material pricing.

**Figure 10:**
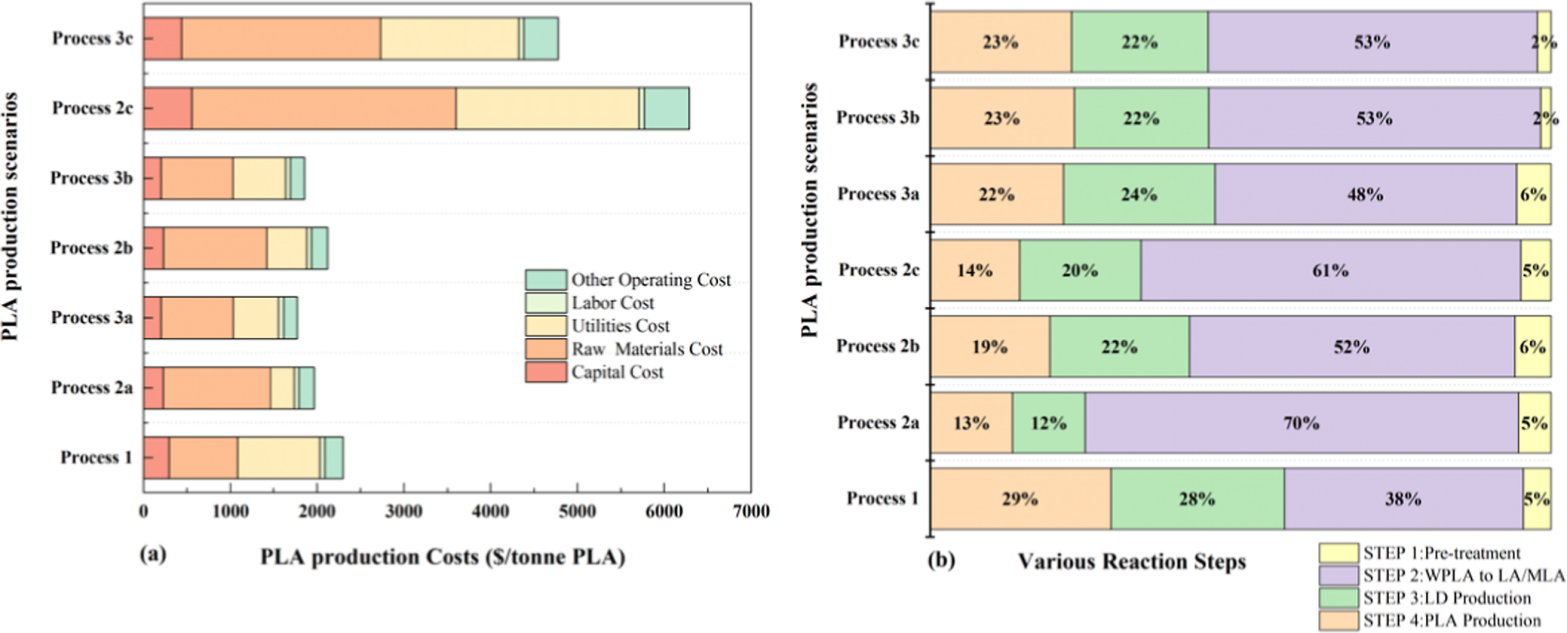
(a) PLA production cost and its breakdown for various scenarios. *Note: Process 1 (the traditional industrial approach) is considered as the base case and (b) Breakdown of the PLA production cost by various reaction steps.

### 3.5 GHG Emissions Analysis

Figure 11 presents the results of GHG emissions analysis for the various processes within the seven considered scenarios. It is evident that Processes 2a and 3a exhibit the lowest carbon emissions, with the production of PLA from LA methyl ester catalyzed by TiO_2_/SiO_2_, yielding 1.89 and 1.55 kg of CO_2_eq per kilogram of PLA, respectively. Process 1, which involves the production of PLA from LA, results in 2.70 kg of CO_2_eq per kilogram of PLA, aligning with the carbon emissions reported in industrial production patents. The carbon emissions of ROP of LD to form PLA are the highest throughout the entire reaction process. This difference is due to the high-purity requirement of the LD purification column in Aspen Plus, which necessitates a more rigorous purification process and significantly increases the emissions related to the reaction. Additionally, the carbon emissions of Processes 2b and 3b are significantly higher than those of Processes 2a and 3a, as well as those of Processes 2c and 3c. Selecting the appropriate catalyst is crucial for reducing carbon emissions in the PLA production process.

**Figure 11:**
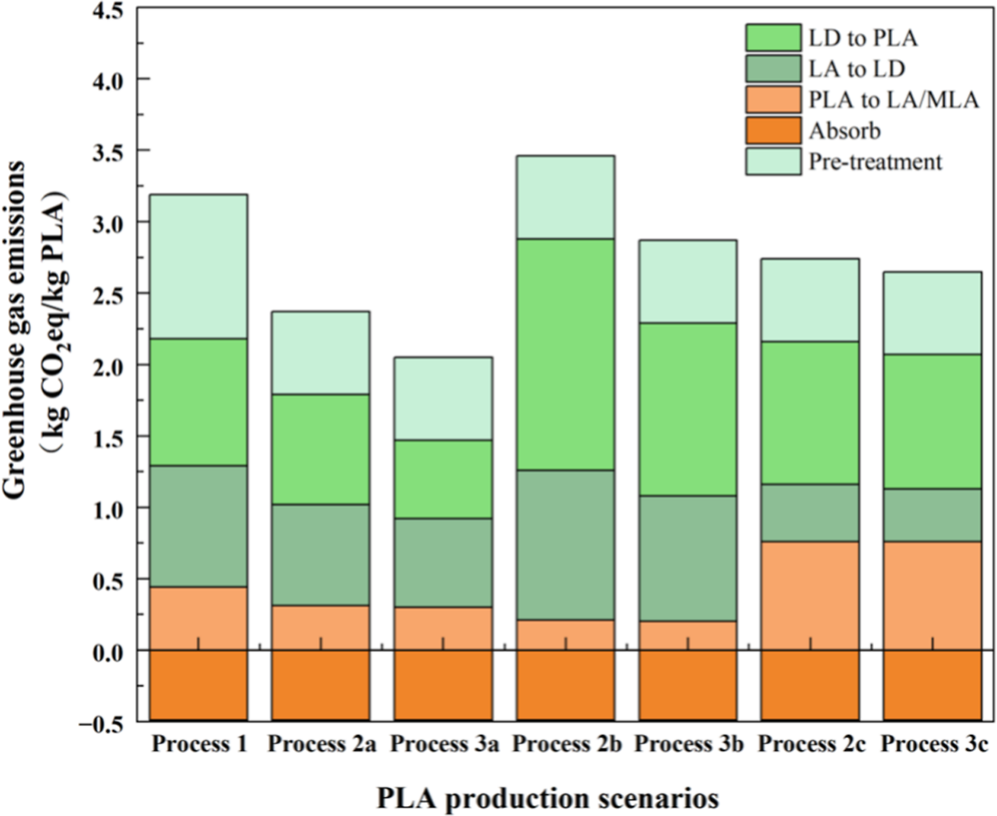
GHG emissions of the PLA recycling processes.

### 3.6 Sensitivity Analysis

Based on the economic analysis and GHG emission analysis results, Process 3a was identified as the most favorable option. Considering the uncertainties parameters, including capital cost, operating cost, and other factors influencing carbon emissions, a sensitivity analysis was conducted to assess the robustness of the results obtained for Process 3a. In this study, the parameters were varied within a range of ±10%, and then the sensitivity ratio was calculated. The outcomes of this sensitivity analysis concerning PLA production costs and GHG emissions are presented in Figure 12. The calculation of the sensitivity ratio is as follows in eq 3

3 $$Sensitivity\;ratio=\frac{\frac{\Delta \;result}{initial\;result}}{\frac{\Delta \;parameter}{initial\;parameter}}$$

**Figure 12:**
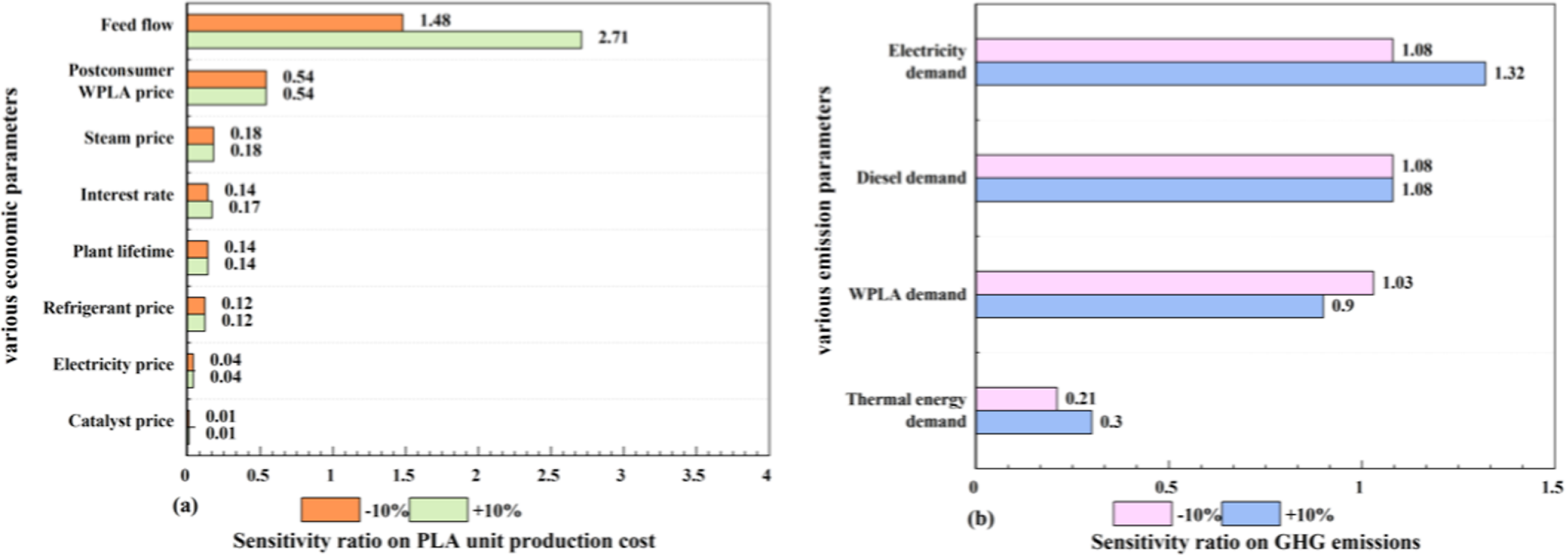
(a) Sensitivity analysis of the PLA production cost and (b) sensitivity analysis of the GHG emissions.

Regarding the unit production cost of PLA (Figure 12a), a sensitivity analysis was conducted across 16 scenarios involving 8 parameters. The feed flow exhibited high sensitivity, with changes of −10% and +10% corresponding to sensitivity ratios of 1.61 and 2.97, respectively. This indicates that fluctuations in the feed flow significantly impact production costs. In contrast, parameters such as catalyst price demonstrated a negligible influence, with sensitivity ratios of only 0.01 for both −10% and +10% variations. With respect to GHG emissions (Figure 12b), a sensitivity analysis was performed across eight scenarios using four parameters. Electricity demand emerged as one of the most influential parameters, with sensitivity ratios of 1.08 and 1.32 corresponding to −10% and +10% changes, respectively. Conversely, the thermal energy demand showed relatively low sensitivity, with sensitivity ratios of 0.21 and 0.3 for −10% and +10% changes, respectively.

### 3.7 Uncertainty Analysis

During the simulation process, there is a certain degree of uncertainty in the input of variables. Figure 13 shows the probability distribution of the unit production cost and GHG emissions of poly­(LA) under random parameter variations. These distributions originated from 1000 Monte Carlo iterations, covering the cumulative effect of ±10% parameter fluctuations and quantifying the likelihood of cost and emission outcomes. For example, in the cost distribution (Figure 13a), compared with process 1, process 3a presents a lower-cost peak. This not only indicates a lower average cost but also implies a smaller variance. On the contrary, as can be seen from Figure 13b, process 3a also has an advantage in terms of GHG emissions, and its distribution is concentrated at lower values, indicating that the environmental performance of process 3a is better under uncertain conditions.

**Figure 13:**
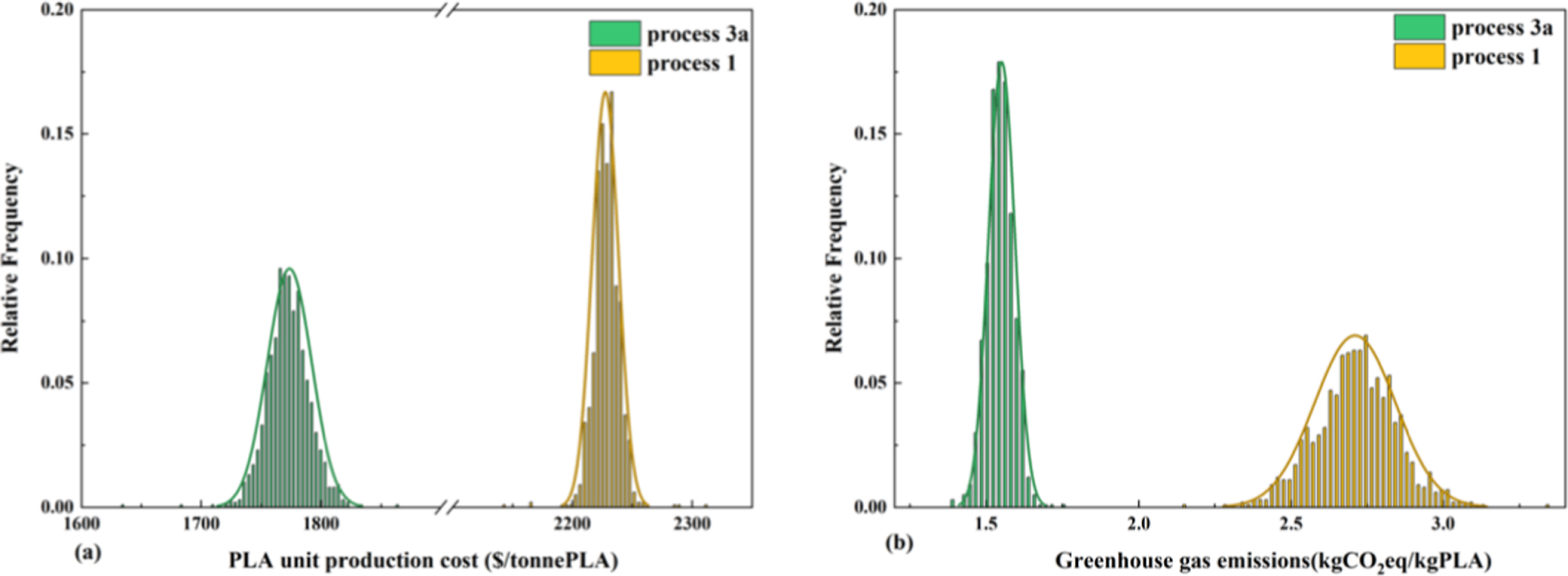
(a) Relative frequency histograms for changes of PLA production cost in various economic parameters and (b) relative frequency histograms for the GHG emissions of the two scenarios.

## 4 Conclusion

In this study, simulations were conducted for recycling WPLA through three distinct processes across seven different scenarios. The results reveal that Process 3a, a one-step LD synthesis process employing a TiO_2_/SiO_2_ catalyst, is the most optimal compared with conventional industrial recycling methods. This approach significantly increased the yield from 59,542 tonnes/year (in traditional industrial scenario) to 95,188 tonnes/year, marking a 59.87% increase. Concurrently, the cost has been reduced by 22.87%, amounting to $1773 per tonne. Additionally, GHG emissions were significantly reduced from 2.70 to 1.55 kg of CO_2_ eq/kg of PLA, indicating a substantial improvement in carbon emission efficiency. A sensitivity analysis of key parameters indicated that the amount of catalyst used, feedstock flow rate, and raw material prices all have a notable impact on PLA production cost. It can be concluded that transforming WPLA into valuable chemicals offers a multifaceted approach that addresses the pressing environmental challenges posed by plastic waste while simultaneously creating economic opportunities. By fostering a transition toward a circular plastic economy, we can achieve a more sustainable and resilient plastic industry that coexists harmoniously with the environment and contributes to global goals of waste reduction and resource conservation.

## Supplementary Material



## Data Availability

Data will be made available on request.

## Notes

Design of the process flow diagram, parameters for techno-economic analysis and GHG analysis, and mass balance for the seven scenarios (PDF)
