# Supplementary material for: Techno-Economic Assessment of a Closed-Loop Circular Economy for Polylactic Acid
Source: ACS Sustain Chem Eng. 2025 Jul 15;13(29):11226–37. doi: 10.1021/acssuschemeng.5c01154 (PMC12308880; doi:10.1021/acssuschemeng.5c01154)
Supplement: Supplementary file 1 [file sc5c01154_si_001.pdf]

Supporting Information

Techno-economic assessment of a closed-loop circular economy for Polylactic Acid (PLA)

Rongrong Zhang [a §], Shuya Jia [b §], Jun Li [b], Yong Xu [c], Hsinghung Chen [a], Xiaolei Zhang [b,]*

[a] *The Institute for Sustainable Development, Macau University of Science and Technology, Macau 999078, China;*

[b] *Department of Chemical and Process Engineering, University of Strathclyde, Glasgow, G1 1XJ, UK*

[c] *Jiangsu Co-Innovation Center of Efficient Processing and Utilization of Forest Resources, College of Chemical Engineering, Nanjing Forestry University, Nanjing 210037, People's Republic of China*

* *Correspondence: xiaolei.zhang@strath.ac.uk*

§ These authors contribute equally to this research.

Process 1: Hydrolysis of PLA Waste

Process 1 is the hydrolysis of WPLA(waste PLA) to lactic acid (LA), which is then polymerized through a two-step process to form lactide (LD), as shown in Figure 3. This intermediate then undergoes ring-opening polymerization to synthesis of the end product new formed PLA.

Reactor Parameter Setting:

Table S1. Types of reactors and other assumption for process 1

Reactor	Type	Gas phase fraction	Reactor	Type	conversion rate(Assumption)
A3	Flash	0.99	A2	RCONV	0.859
A5	Flash	0.89	A4	RCONV	0.97
A7	Flash	0.9	A6	RCONV	0.71
A10	Flash	0.99	A9	RCONV	0.976

Table S2. Specific settings for the separation reactors A8

Performance of Condenser/Top Tray		Performance of Reboiler/Bottom Tray	
Temperature	64 °C	Temperature	200 °C
Heat Duty	-55.32 GJ/hr	Heat Duty	47.88 GJ/hr
Distillate Flow Rate	4482.71kg/hr	Bottoms Flow Rate	7313.89 kg/hr
Reflux Rate	67240kg/hr	Reboiled Vapor Flow Rate	119093 kg/hr
Reflux Ratio	15	Reboil Ratio	15
Distillate to Feed Ratio	0.38	Bottoms Withdrawal to Feed Ratio	0.62

Mass balance for process:

Table S3. Mass Balance for Process 1

Stream	1	2	3	4	5	6	7	8	9
Temperature(°C)	160.00	25.00	160.00	180.00	180.00	180.00	200.00	200.00	200.00
Pressure(bar)	1.00	1.00	1.00	1.00	0.23	0.23	0.33	0.40	0.40
flow rate(kg/h)	20000.00	5000.00	25000.00	25000.00	2842.61	22157.39	22157.39	17164.41	4992.98
Mass Frac									
WATER	0.0000	1.0000	0.2000	0.0283	0.0000	0.0320	0.1963	0.0000	0.8711
L-LA	0.0000	0.0000	0.0000	0.8589	0.0079	0.9680	0.0290	0.0000	0.1289
L-LD	0.0000	0.0000	0.0000	0.0000	0.0000	0.0000	0.0000	0.0000	0.0000

M-LD	0.0000	0.0000	0.0000	0.0000	0.0000	0.0000	0.0000	0.0000	0.0000
Oligmer	0.0000	0.0000	0.0000	0.0000	0.0000	0.0000	0.7747	1.0000	0.0000
WPLA	1.0000	0.0000	0.8000	0.1128	0.9920	0.0000	0.0000	0.0000	0.0000
PLA	0.0000	0.0000	0.0000	0.0000	0.0000	0.0000	0.0000	0.0000	0.0000
**Stream**	**10**	**11**	**12**	**13**	**14**	**15**	**16**	**17**	**18**
Temperature(˚C)	220.00	250.00	250.00	200.81	63.99	25.00	80.00	220.00	220.00
Pressure(bar)	0.01	0.50	0.50	0.10	0.10	1.00	1.00	0.10	0.10
flow rate(kg/h)	17164.41	5367.81	11796.60	7313.89	4482.71	1.80	7315.69	6796.98	518.71
Mass Frac									
WATER	0.0215	0.0000	0.0313	0.0000	0.0823	1.0000	0.0002	0.0000	0.0022
L-LA	0.0000	0.0000	0.0000	0.0000	0.0000	0.0000	0.0000	0.0000	0.0000
L-LD	0.5852	0.0616	0.8235	0.9521	0.6137	0.0000	0.0228	0.0000	0.3222
M-LD	0.1033	0.0111	0.1452	0.0479	0.3040	0.0000	0.0479	0.0000	0.6757
Oligmer	0.2900	0.9273	0.0000	0.0000	0.0000	0.0000	0.0000	0.0000	0.0000
WPLA	0.0000	0.0000	0.0000	0.0000	0.0000	0.0000	0.0000	0.0000	0.0000
PLA	0.0000	0.0000	0.0000	0.0000	0.0000	0.0000	0.9291	1.0000	0.0000

Process 2: Alcoholysis of PLA Waste

Process 2 is the alcoholysis of WPLA to produce Methyl lactate(MLA) as shown in Figure 4, which is directly converted to LD in a one-step gas-phase reaction[1]. Mirroring Process 1, the LD then undergoes ring-opening polymerization to form PLA. Notably, the different catalysts TiO_2/SiO_2, TiO_2/MCM-41, and TiO_2, were used in LD production and defined as process 2a, 2b, 2c.

Reactor Parameter Setting:

Table S4. Types of reactors and other assumption for process 2

**Reactor**	**Type**	**Gas phase fraction**	**Reactor**	**Type**	**conversion rate(Assumption)**
B3	Flash	0.99	B2	RCONV	0.97
B6	Flash	0.99	B5	RCONV	Process 2a:0.42<br>Process 2b:0.46<br>Process 2c:0.32
B9	Flash	0.99	B8	RCONV	0.97

Table S5. Specific settings for the separation reactors B7

Performance of Condenser/Top Tray		Performance of Reboiler/Bottom Tray	
Temperature	20 ˚C	Temperature	140 ˚C
Heat Duty	-13.96GJ/hr	Heat Duty	14.14 GJ/hr
Distillate Flow Rate	3300kg/hr	Bottoms Flow Rate	7500kg/hr
Reflux Rate	20166kg/hr	Reboiled Vapor Flow Rate	32956 kg/hr
Reflux Ratio	6	Reboil Ratio	4.4
Distillate to Feed Ratio	0.30	Bottoms Withdrawal to Feed Ratio	0.69

Mass balance for process：

Table S6. Mass Balance for Process 2a (TiO2 / SiO2)

Stream	21	22	23	24	25	26	27	28	29
Temperature(˚C)	160.00	80.00	160.00	130.00	140.00	140.00	210.00	138.96	220.00
Pressure(bar)	1.00	1.00	1.00	1.00	1.03	1.03	1.00	0.24	0.24
flow rate(kg/h)	20000.00	10000.00	30000.00	30000.00	29368.39	631.61	0.00	29368.39	29368.39
Mass Frac									
WATER	0.0000	0.0000	0.0000	0.0001	0.0001	0.0000	0.0000	0.0001	0.0001
L-LD	0.0000	0.0000	0.0000	0.0000	0.0000	0.0000	0.0000	0.0000	0.2632
M-LD	0.0000	0.0000	0.0000	0.0000	0.0000	0.0000	0.0000	0.0000	0.0139
PLA	1.0000	0.0000	0.6667	0.0200	0.0000	0.9500	0.0000	0.0000	0.0000
CH_3OH	0.0000	1.0000	0.3333	0.0458	0.0468	0.0002	0.0000	0.0468	0.1700
MLA	0.0000	0.0000	0.0000	0.9340	0.9531	0.0498	0.0000	0.9531	0.5528
N_2	0.0000	0.0000	0.0000	0.0000	0.0000	0.0000	1.0000	0.0000	0.0000
PLA	0.0000	0.0000	0.0000	0.0000	0.0000	0.0000	0.0000	0.0000	0.0000

Stream	30	31	32	33	34	35	36	37
Temperature(˚C)	90.00	90.00	139.02	20.84	25.00	80.00	220.00	220.00
Pressure(bar)	0.10	0.10	0.01	0.01	1.00	1.00	0.10	0.10
flow rate(kg/h)	10808.26	18560.13	7507.67	3300.59	1.80	7509.47	7286.35	223.12
Mass Frac								
WATER	0.0000	0.0002	0.0000	0.0000	1.0000	0.0001	0.0000	0.0048
L-LD	0.6958	0.0113	0.9943	0.0168	0.0000	0.0239	0.0000	0.8030
M-LD	0.0355	0.0012	0.0057	0.1034	0.0000	0.0057	0.0000	0.1922
PLA	0.0000	0.0000	0.0000	0.0000	0.0000	0.0000	0.0000	0.0000

CH_3OH	0.0055	0.2659	0.0000	0.0179	0.0000	0.0000	0.0000	0.0000
MLA	0.2632	0.7214	0.0000	0.8619	0.0000	0.0000	0.0000	0.0000
N_2	0.0000	0.0000	0.0000	0.0000	0.0000	0.0000	0.0000	0.0000
PLA	0.0000	0.0000	0.0000	0.0000	0.0000	0.9703	1.0000	0.0000

Table S7. Mass Balance for Process 2b (TiO2/MCM-41)

Stream	21	22	23	24	25	26	27	28	29
Temperature(˚C)	160.00	80.00	160.00	130.00	140.00	140.00	210.00	138.96	220.00
Pressure(bar)	1.00	1.00	1.00	1.00	1.03	1.03	1.00	0.24	0.24
flow rate(kg/h)	20000.00	10000.00	30000.00	30000.00	29368.39	631.61	0.00	29368.39	29368.39
Mass Frac									
WATER	0.0000	0.0000	0.0000	0.0001	0.0001	0.0000	0.0000	0.0001	0.0001
L-LD	0.0000	0.0000	0.0000	0.0000	0.0000	0.0000	0.0000	0.0000	0.0000
M-LD	0.0000	0.0000	0.0000	0.0000	0.0000	0.0000	0.0000	0.0000	0.2883
PLA	1.0000	0.0000	0.6667	0.0200	0.0000	0.9500	0.0000	0.0000	0.0152
CH_3OH	0.0000	1.0000	0.3333	0.0458	0.0468	0.0002	0.0000	0.0468	0.0000
MLA	0.0000	0.0000	0.0000	0.9340	0.9531	0.0498	0.0000	0.9531	0.0000
N_2	0.0000	0.0000	0.0000	0.0000	0.0000	0.0000	1.0000	0.0000	0.1818
PLA	0.0000	0.0000	0.0000	0.0000	0.0000	0.0000	0.0000	0.0000	0.5147

Stream	30	31	32	33	34	35	36	37
Temperature(˚C)	90.00	90.00	139.05	20.27	25.00	80.00	220.00	220.00
Pressure(bar)	0.10	0.10	0.01	0.01	1.00	1.00	0.10	0.10
flow rate(kg/h)	11504.69	17863.70	7937.44	3567.26	1.80	7939.24	7725.46	213.78
Mass Frac								
WATER	0.0000	0.0002	0.0000	0.0000	1.0000	0.0001	0.0000	0.0048
L-LD	0.0000	0.0000	0.0000	0.0000	0.0000	0.0000	0.0000	0.0000
M-LD	0.7169	0.0122	0.9971	0.0935	0.0000	0.0239	0.0000	0.8885
PLA	0.0367	0.0013	0.0029	0.1119	0.0000	0.0029	0.0000	0.1067
CH_3OH	0.0000	0.0000	0.0000	0.0000	0.0000	0.0000	0.0000	0.0000
MLA	0.0000	0.0000	0.0000	0.0000	0.0000	0.0000	0.0000	0.0000
N_2	0.0058	0.2951	0.0000	0.0186	0.0000	0.0000	0.0000	0.0000
PLA	0.2406	0.6911	0.0000	0.7760	0.0000	0.0000	0.0000	0.0000

Table S8. Mass Balance for Process 2c(TiO_2)

Stream	21	22	23	24	25	26	27	28	29
Temperature(˚C)	160.00	80.00	160.00	130.00	140.00	140.00	210.00	138.96	220.00
Pressure(bar)	1.00	1.00	1.00	1.00	1.03	1.03	1.00	0.24	0.24

flow rate(kg/h)	20000.00	10000.00	30000.00	30000.00	29368.39	631.61	0.00	29368.39	29368.39
Mass Frac									
WATER	0.0000	0.0000	0.0000	0.0001	0.0001	0.0000	0.0000	0.0001	0.0001
L-LD	0.0000	0.0000	0.0000	0.0000	0.0000	0.0000	0.0000	0.0000	0.0000
M-LD	0.0000	0.0000	0.0000	0.0000	0.0000	0.0000	0.0000	0.0000	0.1034
PLA	1.0000	0.0000	0.6667	0.0200	0.0000	0.9500	0.0000	0.0000	0.1077
CH_3OH	0.0000	1.0000	0.3333	0.0458	0.0468	0.0002	0.0000	0.0468	0.0000
MLA	0.0000	0.0000	0.0000	0.9340	0.9531	0.0498	0.0000	0.9531	0.0000
N_2	0.0000	0.0000	0.0000	0.0000	0.0000	0.0000	1.0000	0.0000	0.1407
PLA	0.0000	0.0000	0.0000	0.0000	0.0000	0.0000	0.0000	0.0000	0.6481

Stream	30	31	32	33	34	35	36	37
Temperature(°C)	90.00	90.00	139.05	20.27	25.00	80.00	220.00	220.00
Pressure(bar)	0.10	0.10	0.01	0.01	1.00	1.00	0.10	0.10
flow rate(kg/h)	17993.75	11374.65	12852.03	5141.71	1.80	12853.83	2956.47	9897.37
Mass Frac								
WATER	0.0000	0.0002	0.0000	0.0001	1.0000	0.0001	0.0000	0.0002
L-LD	0.0000	0.0000	0.0000	0.0000	0.0000	0.0000	0.0000	0.0000
M-LD	0.1683	0.0008	0.2357	0.0000	0.0000	0.0057	0.0000	0.0073
PLA	0.1742	0.0025	0.2438	0.0000	0.0000	0.2438	0.0000	0.3166
CH_3OH	0.0000	0.0000	0.0000	0.0000	0.0000	0.0000	0.0000	0.0000
MLA	0.0000	0.0000	0.0000	0.0000	0.0000	0.0000	0.0000	0.0000
N_2	0.0147	0.3400	0.0000	0.0514	0.0000	0.0000	0.0000	0.0000
PLA	0.6428	0.6565	0.5205	0.9485	0.0000	0.5204	0.0000	0.6759

Process 3: Alcoholysis of PLA Waste

As shown in Figure 5, Process 3 represents our own experimental innovation, which is an improvement on Process 2. In Process 3, the synthesis of MLA and PLA mirrors that of Process 2, with the recycling of unreacted MLA was added to enhance the yield of LD due to the conversion efficiency of MLA to LD is suboptimal. Additionally, Process 3 is further differentiated by the employment of distinct catalytic systems: TiO_2/SiO_2, TiO_2/MCM-41, and TiO_2, which are respectively termed as Process 3a, 3b, and 3c.

Reactor Parameter Setting：

Table S9. Types of reactors and other assumption for process 3

Reactor	Type	Gas phase fraction	Reactor	Type	conversion rate(Assumption)
B3	Flash	0.99	B2	RCONV	0.97

B6	Flash	0.99	B5	RCONV	Process 3a:0.42<br>Process 3b:0.46<br>Process 3c:0.32
B9	Flash	0.99	B8	RCONV	0.97

Table S10. Specific settings for the separation reactors B7

**Performance of Condenser/Top Tray**		**Performance of Reboiler/Bottom Tray**	
Temperature	20 °C	Temperature	130 °C
Heat Duty	-22.40GJ/hr	Heat Duty	22.68GJ/hr
Distillate Flow Rate	5200kg/hr	Bottoms Flow Rate	11660kg/hr
Reflux Rate	31600kg/hr	Reboiled Vapor Flow Rate	52800kg/hr
Reflux Ratio	6.11	Reboil Ratio	4.5
Distillate to Feed Ratio	0.30	Bottoms Withdrawal to Feed Ratio	0.69

Mass balance for process：

Table S11. Mass Balance for Process 3a(TiO_2 / SiO_2)

**Stream**	**41**	**42**	**43**	**44**	**45**	**46**	**47**	**48**	**49**	**50**
Temperature(°C)	160.00	80.00	160.00	130.00	140.00	140.00	210.00	138.96	220.00	90.00
Pressure(bar)	1.00	1.00	1.00	1.00	1.03	1.03	1.00	0.24	0.24	0.10
flow rate(kg/h)	20000.00	10000.00	30000.00	30000.00	29368.39	631.61	0.00	29368.39	42757.39	26467.40
Mass Frac										
WATER	0.0000	0.0000	0.0000	0.0001	0.0001	0.0000	0.0000	0.0001	0.0001	0.0001
L-LD	0.0000	0.0000	0.0000	0.0000	0.0000	0.0000	0.0000	0.0000	0.2673	0.0109
M-LD	0.0000	0.0000	0.0000	0.0000	0.0000	0.0000	0.0000	0.0000	0.0141	0.0012
WPLA	1.0000	0.0000	0.6667	0.0200	0.0000	0.9500	0.0000	0.0000	0.0000	0.0000
CH_3OH	0.0000	1.0000	0.3333	0.0458	0.0468	0.0002	0.0000	0.0468	0.1573	0.2508
MLA	0.0000	0.0000	0.0000	0.9340	0.9531	0.0498	0.0000	0.9531	0.5613	0.7370
N_2	0.0000	0.0000	0.0000	0.0000	0.0000	0.0000	1.0000	0.0000	0.0000	0.0000
PLA	0.0000	0.0000	0.0000	0.0000	0.0000	0.0000	0.0000	0.0000	0.0000	0.0000
**Stream**	**51**	**52**	**53**	**54**	**55**	**56**	**57**	**58**	**59**	**60**
Temperature(°C)	90.00	90.00	90.00	90.00	138.89	21.16	25.00	80.00	220.00	220.00
Pressure(bar)	0.10	0.10	0.10	0.10	0.01	0.01	1.00	1.00	0.10	0.10
flow rate(kg/h)	13078.40	311.00	13389.00	16289.99	11357.73	4932.26	1.80	11359.53	10866.25	493.28
Mass Frac										

WATER	0.0002	0.0000	0.0000	0.0000	0.0000	0.0000	1.0000	0.0001	0.0000	0.0014
L-LD	0.0220	0.4000	0.0000	0.6839	0.9802	0.0018	0.0000	0.0235	0.0000	0.5416
M-LD	0.0024	0.0924	0.0000	0.0350	0.0198	0.0699	0.0000	0.0198	0.0000	0.4569
WPLA	0.0000	0.0000	0.0000	0.0000	0.0000	0.0000	0.0000	0.0000	0.0000	0.0000
CH_3OH	0.5076	0.5076	0.0000	0.0053	0.0000	0.0174	0.0000	0.0000	0.0000	0.0000
MLA	0.4678	0.0000	1.0000	0.2758	0.0000	0.9109	0.0000	0.0000	0.0000	0.0000
N_2	0.0000	0.0000	0.0000	0.0000	0.0000	0.0000	0.0000	0.0000	0.0000	0.0000
PLA	0.0000	0.0000	0.0000	0.0000	0.0000	0.0000	0.0000	0.9566	1.0000	0.0000

Table S12. Mass Balance for Process 3b(TiO_2/MCM-41)

**Stream**	**41**	**42**	**43**	**44**	**45**	**46**	**47**	**48**	**49**	**50**
Temperature(˚C)	160.00	80.00	160.00	130.00	140.00	140.00	210.00	138.96	220.00	90.00
Pressure(bar)	1.00	1.00	1.00	1.00	1.03	1.03	1.00	0.24	0.24	0.10
flow rate(kg/h)	20000.00	10000.00	30000.00	30000.00	29368.39	631.61	0.00	29368.39	41714.85	24870.05
Mass Frac										
WATER	0.0000	0.0000	0.0000	0.0001	0.0001	0.0000	0.0000	0.0001	0.0000	0.0001
L-LD	0.0000	0.0000	0.0000	0.0000	0.0000	0.0000	0.0000	0.0000	0.7069	0.0118
M-LD	0.0000	0.0000	0.0000	0.0000	0.0000	0.0000	0.0000	0.0000	0.0362	0.0013
WPLA	1.0000	0.0000	0.6667	0.0200	0.0000	0.9500	0.0000	0.0000	0.0000	0.0000
CH_3OH	0.0000	1.0000	0.3333	0.0458	0.0468	0.0002	0.0000	0.0468	0.0056	0.2811
MLA	0.0000	0.0000	0.0000	0.9340	0.9531	0.0498	0.0000	0.9531	0.2512	0.7057
N_2	0.0000	0.0000	0.0000	0.0000	0.0000	0.0000	1.0000	0.0000	0.0000	0.0000
PLA	0.0000	0.0000	0.0000	0.0000	0.0000	0.0000	0.0000	0.0000	0.0000	0.0000
**Stream**	**51**	**52**	**53**	**54**	**55**	**56**	**57**	**58**	**59**	**60**
Temperature(˚C)	90.00	90.00	90.00	90.00	139.04	20.54	25.00	80.00	220.00	220.00
Pressure(bar)	0.10	0.10	0.10	0.10	0.01	0.01	1.00	1.00	0.10	0.10
flow rate(kg/h)	12523.55	177.09	12346.46	16844.80	11658.64	5186.17	1.80	11660.44	11340.82	319.62
Mass Frac										
WATER	0.0002	0.0000	0.0000	0.0000	0.0000	0.0000	1.0000	0.0001	0.0000	0.0021
L-LD	0.0234	0.5000	0.0000	0.7069	0.9966	0.0558	0.0000	0.0239	0.0000	0.8724
M-LD	0.0026	0.0024	0.0000	0.0362	0.0034	0.1099	0.0000	0.0034	0.0000	0.1255
WPLA	0.0000	0.0000	0.0000	0.0000	0.0000	0.0000	0.0000	0.0000	0.0000	0.0000
CH_3OH	0.5583	0.4976	0.0000	0.0056	0.0000	0.0183	0.0000	0.0000	0.0000	0.0000
MLA	0.4155	0.0000	1.0000	0.2512	0.0000	0.8159	0.0000	0.0000	0.0000	0.0000
N_2	0.0000	0.0000	0.0000	0.0000	0.0000	0.0000	0.0000	0.0000	0.0000	0.0000
PLA	0.0000	0.0000	0.0000	0.0000	0.0000	0.0000	0.0000	0.9726	1.0000	0.0000

Table S13. Mass Balance for Process 3c(TiO_2)

Stream	41	42	43	44	45	46	47	48	49	50
Temperature(˚C)	160.00	80.00	160.00	130.00	140.00	140.00	210.00	138.96	220.00	70.00
Pressure(bar)	1.00	1.00	1.00	1.00	1.03	1.03	1.00	0.24	0.24	0.10
flow rate(kg/h)	20000.00	10000.00	30000.00	30000.00	29368.39	631.61	0.00	29368.39	38586.32	13941.29
Mass Frac										
WATER	0.0000	0.0000	0.0000	0.0001	0.0001	0.0000	0.0000	0.0001	0.0001	0.0002
L-LD	0.0000	0.0000	0.0000	0.0000	0.0000	0.0000	0.0000	0.0000	0.1047	0.0008
M-LD	0.0000	0.0000	0.0000	0.0000	0.0000	0.0000	0.0000	0.0000	0.1089	0.0024
WPLA	1.0000	0.0000	0.6667	0.0200	0.0000	0.9500	0.0000	0.0000	0.0000	0.0000
CH_3OH	0.0000	1.0000	0.3333	0.0458	0.0468	0.0002	0.0000	0.0468	0.1306	0.3357
MLA	0.0000	0.0000	0.0000	0.9340	0.9531	0.0498	0.0000	0.9531	0.6557	0.6610
N_2	0.0000	0.0000	0.0000	0.0000	0.0000	0.0000	1.0000	0.0000	0.0000	0.0000
PLA	0.0000	0.0000	0.0000	0.0000	0.0000	0.0000	0.0000	0.0000	0.0000	0.0000
**Stream**	**51**	**52**	**53**	**54**	**55**	**56**	**57**	**58**	**59**	**60**
Temperature(˚C)	70.00	90.00	70.00	70.00	51.52	10.18	25.00	80.00	220.00	220.00
Pressure(bar)	0.10	0.10	0.10	0.10	0.01	0.01	1.00	1.00	0.10	0.10
flow rate(kg/h)	4726.10	311.00	9217.93	24645.03	17576.79	7068.24	1.80	17578.59	3931.46	13647.14
Mass Frac										
WATER	0.0005	0.0000	0.0000	0.0000	0.0000	0.0001	1.0000	0.0001	0.0000	0.0001
L-LD	0.0023	0.4000	0.0000	0.1634	0.2292	0.0000	0.0000	0.0055	0.0000	0.0071
M-LD	0.0070	0.0924	0.0000	0.1692	0.2373	0.0000	0.0000	0.2372	0.0000	0.3056
WPLA	0.0000	0.0000	0.0000	0.0000	0.0000	0.0000	0.0000	0.0000	0.0000	0.0000
CH_3OH	0.9902	0.5076	0.0000	0.0146	0.0000	0.0510	0.0000	0.0000	0.0000	0.0000
MLA	0.0000	0.0000	1.0000	0.6527	0.5336	0.9489	0.0000	0.5335	0.0000	0.6872
N_2	0.0000	0.0000	0.0000	0.0000	0.0000	0.0000	0.0000	0.0000	0.0000	0.0000
PLA	0.0000	0.0000	0.0000	0.0000	0.0000	0.0000	0.0000	0.2237	1.0000	0.0000

Cost for all scenarios:

Table S14. Summary of the capital cost for scenarios($/tonne PLA)

	Process1	Process 2a	Processo 3a	Process 2b	Process 3b	Process 2c	Process 3c
Annual production rate （tonnes/year）	56564.48	60636.98	90428.92	64291.25	93177.08	24603.72	32717.57
**Direct cost**							
Equipment purchase cost(EC)	43.5728	33.7711	30.3738	34.4745	30.6216	79.4366	62.9099
Installation	83.5381	62.8501	53.7525	62.1267	53.3787	167.1157	131.7497
piping	13.5076	10.4690	9.4159	10.6871	9.4927	24.6253	19.5021
instrumentation	18.7363	14.5216	13.0607	14.8240	13.1673	34.1577	27.0513

electrical	4.3573	3.3771	3.0374	3.4475	3.0622	7.9437	6.2910
buildings	6.5359	5.0657	4.5561	5.1712	4.5932	11.9155	9.4365
yard improvement	5.2287	4.0525	3.6449	4.1369	3.6746	9.5324	7.5492
service facilities	23.9650	18.5741	16.7056	18.9610	16.8419	43.6901	34.6004
Land	2.6144	2.0263	1.8224	2.0685	1.8373	4.7662	3.7746
**Indirect Cost**							
Engineering and supervision	13.9433	10.8067	9.7196	11.0318	9.7989	25.4197	20.1312
construction expense	14.8148	11.4822	10.3271	11.7213	10.4114	27.0084	21.3894
legal expenses	1.7429	1.3508	1.2150	1.3790	1.2249	3.1775	2.5164
Contractor's fee	8.2788	6.4165	5.7710	6.5502	5.8181	15.0929	11.9529
Contingency	16.1219	12.4953	11.2383	12.7556	11.3300	29.3915	23.2767
fixed capital investment=Direct cost+ Indirect cost	256.9579	197.2589	174.6402	199.3353	175.2528	483.2732	382.1311
working capital	38.5437	29.5888	26.1960	29.9003	26.2879	72.4910	57.3197
**Annual capital cost/ton PLA**	295.5015	226.8478	200.8362	229.2356	201.5407	555.7642	439.4508

Table S15. Summary of the operating cost for scenarios($/tonne PLA)

	Process 1	Process 2a	Process 3a	Process 2b	Process3b	Process 2c	Process 3c
Annual production rate （ton/year）	56564.48	60636.98	90428.92	64291.25	93177.08	24603.72	32717.57
**Raw materials cost**							
feed stock cost	770.2228	1235.4380	829.1042	1165.3345	804.7118	3041.7561	2287.9248
catalyst cost	18.7213	2.3741	2.0423	23.4911	23.5515	7.0926	5.3337
**Labour cost**	**58.1880**	**53.7037**	**58.1880**	**55.7785**	**54.5901**	**61.9622**	**59.4779**
**Utility cost**							
electrical cost	75.1738	55.6108	45.9634	60.0331	44.9427	123.9382	96.0182
Steam cost	588.1329	217.1223	301.5693	255.0602	353.5912	1287.2980	972.4512
Cooling water cost	285.3764	1.7824	177.7744	148.2477	209.9198	696.2375	523.6009
**Fixed Expenses**	**208.0441**	**174.2799**	**157.9768**	**185.1368**	**162.9399**	**512.5155**	**394.2404**
**All operating cost/tonne PLA**	2003.8593	1740.3112	1572.6184	1893.0818	1654.2470	5730.8002	4339.0470

Table S16. Summary of the capital and operating cost steps for scenarios($/year)

	ALL	WPLA-LA	LA-LD	LD-PLA	Pre-treatment
process 1					
capital cost	16714891	917663	12292000	878873	2626355
raw materials cost	44626214	43879201	619662	10063	117288
labor cost	3291374	902448	902448	902448	584030
Utility cost	53661762	721964	17940697	33347064	1652037

other operating cost	11767907	3544005	4556519	2760121	907262
All annual cost	130062148	49965282	36311329	37898571	5886966
process 2a					
capital cost	13755365	1009987	9052321	877611	2815446
raw materials cost	75057186	74835710	84958	10786	125732
labor cost	3256430	789105	1052141	789105	626079
Utility cost	16645796	805043	1174636	12895137	1770980
other operating cost	10567810	5726239	2566889	1302100	972582
All annual cost	119282588	83166087	13930946	15874741	6310814
process 2b					
capital cost	14737840	1009987	9865125	877611	2985117
raw materials cost	76431079	74835715	1290562	171493	133309
labor cost	3586069	876678	1168904	876678	663809
Utility cost	29788770	938853	10775584	16196626	1877707
other operating cost	11902673	5755791	3551049	1564639	1031194
All annual cost	136446430	83417026	26651225	19687048	6691131
process 2c					
capital cost	13673867	1009987	10640917	880582	1142381
raw materials cost	75013020	74835715	60660	65629	51016
labor cost	1524500	381139	508186	381139	254036
Utility cost	51851696	830982	18235236	32066894	718584
other operating cost	12609789	5634019	4026480	2554660	394630
All annual cost	154672871	82691844	33471481	35948906	2560640
process 3a					
capital cost	18161405	1009987	12214458	738240	4198720
raw materials cost	75159691	74830893	125596	15696	187506
labor cost	5261878	1298459	1731279	1298459	933681
Utility cost	47502955	938853	17317557	26605455	2641090
other operating cost	14285668	5852674	4637423	2345143	1450428
All annual cost	160371597	83930869	36026315	31002995	9411418
process 3b					
capital cost	18778979	1009987	12704431	738240	4326321
raw materials cost	77175163	74835715	1894496	251748	193204
labor cost	5086543	1237346	1649795	1237346	962056
Utility cost	56693942	938853	20801015	32232721	2721353
other operating cost	15182265	5838925	5107345	2741489	1494506
All annual cost	172916892	83860828	42157084	37201546	9697434
process 3c					
capital cost	14377761	1009987	11283609	565050	1519115

raw materials cost	75029844	74835715	60660	65629	67840
labor cost	1945974	381139	508186	381139	675510
Utility cost	52088671	830982	18235236	32066894	955559
other operating cost	12898588	5634019	4185244	2476714	602611
All annual cost	156340838	82691844	34272936	35555427	3820631

Table S17. Assumptions and data used for life cycle inventories (scope 3)

**Items**	**Estimation assumption**	**Data source**
**Input flows**		
Postconsumer PLA waste	2120kg	Assumed
Transport of pre-treatment pla recycling	25 km	Assumed
Diesel demand for PLA waste collection and transportation	1.98kg/ tonnes pla	Amount of fuel calculated with GaBi
Water demand for washing	200kg/tonnes pla	Measured
Washing agent demand (2% aq. NaOH solution at 70°C)	4kg/ tonnes pla	Mega et al., 2019[2]
Thermal energy demand for drying	43kwh/ tonnes pla	Measured
Electricity demand for shredding	63.53kwh/tonnes pla	Measured
Thermal energy demand for the entire downstream processing：process1	15960 MJ/tonnes pla	Mega et al., 2021[3]
Thermal energy demand for the entire downstream processing：process2 process3	8523 MJ/tonnes pla	Mega et al., 2021[3]
**Output flows**		
Residues after sorting stage	25.44kg	Mega et al., 2021[3]
Residues after washing stage	57.24kg	Mega et al., 2021[3]
Residues after shredding stage	38.15kg	Mega et al., 2021[3]
Net amount of PLA waste after pre-treatment	2000kg	Mega et al., 2021[3]

References

(1) De Clercq, R.; Dusselier, M.; Makshina, E.; Sels, B. F. Catalytic Gas-Phase Production of Lactide from Renewable Alkyl Lactates. *Angew Chem Int Ed* 2018, *57* (12), 3074–3078. https://doi.org/10.1002/anie.201711446.

(2) Maga, D.; Hiebel, M.; Thonemann, N. Life Cycle Assessment of Recycling Options for Polylactic Acid. *Resources, Conservation and Recycling* 2019, *149*, 86–96. https://doi.org/10.1016/j.resconrec.2019.05.018.

(3) Aryan, V.; Maga, D.; Majgaonkar, P.; Hanich, R. Valorisation of Polylactic Acid (PLA) Waste: A Comparative Life Cycle Assessment of Various Solvent-Based Chemical Recycling Technologies. *Resources, Conservation and Recycling* 2021, *172*, 105670. https://doi.org/10.1016/j.resconrec.2021.105670.
